# The Implementation of Recommender Systems for Mental Health Recovery Narratives: Evaluation of Use and Performance

**DOI:** 10.2196/45754

**Published:** 2024-03-29

**Authors:** Emily Slade, Stefan Rennick-Egglestone, Fiona Ng, Yasuhiro Kotera, Joy Llewellyn-Beardsley, Chris Newby, Tony Glover, Jeroen Keppens, Mike Slade

**Affiliations:** 1 School of Health Sciences Institute of Mental Health University of Nottingham Nottingham United Kingdom; 2 Department of Computer Science University of Oxford Oxford United Kingdom; 3 Center for Infectious Disease Education and Research, Osaka University Osaka Japan; 4 School of Medicine, University of Nottingham Nottingham United Kingdom; 5 DRT Software Ltd. Nottingham United Kingdom; 6 Department of Informatics King's College London London United Kingdom; 7 Nord University Faculty of Nursing and Health Sciences Namsos Norway

**Keywords:** recommender system, mean absolute error, precision, intralist diversity, item space coverage, fairness across users, psychosis, Narrative Experiences Online trial, NEON trial, lived experience narrative, recovery story

## Abstract

**Background:**

Recommender systems help narrow down a large range of items to a smaller, personalized set. NarraGive is a first-in-field hybrid recommender system for mental health recovery narratives, recommending narratives based on their content and narrator characteristics (using content-based filtering) and on narratives beneficially impacting other similar users (using collaborative filtering). NarraGive is integrated into the Narrative Experiences Online (NEON) intervention, a web application providing access to the NEON Collection of recovery narratives.

**Objective:**

This study aims to analyze the 3 recommender system algorithms used in NarraGive to inform future interventions using recommender systems for lived experience narratives.

**Methods:**

Using a recently published framework for evaluating recommender systems to structure the analysis, we compared the content-based filtering algorithm and collaborative filtering algorithms by evaluating the accuracy (how close the predicted ratings are to the true ratings), precision (the proportion of the recommended narratives that are relevant), diversity (how diverse the recommended narratives are), coverage (the proportion of all available narratives that can be recommended), and unfairness (whether the algorithms produce less accurate predictions for disadvantaged participants) across gender and ethnicity. We used data from all participants in 2 parallel-group, waitlist control clinical trials of the NEON intervention (NEON trial: N=739; NEON for other [eg, nonpsychosis] mental health problems [NEON-O] trial: N=1023). Both trials included people with self-reported mental health problems who had and had not used statutory mental health services. In addition, NEON trial participants had experienced self-reported psychosis in the previous 5 years. Our evaluation used a database of Likert-scale narrative ratings provided by trial participants in response to validated narrative feedback questions.

**Results:**

Participants from the NEON and NEON-O trials provided 2288 and 1896 narrative ratings, respectively. Each rated narrative had a median of 3 ratings and 2 ratings, respectively. For the NEON trial, the content-based filtering algorithm performed better for coverage; the collaborative filtering algorithms performed better for accuracy, diversity, and unfairness across both gender and ethnicity; and neither algorithm performed better for precision. For the NEON-O trial, the content-based filtering algorithm did not perform better on any metric; the collaborative filtering algorithms performed better on accuracy and unfairness across both gender and ethnicity; and neither algorithm performed better for precision, diversity, or coverage.

**Conclusions:**

Clinical population may be associated with recommender system performance. Recommender systems are susceptible to a wide range of undesirable biases. Approaches to mitigating these include providing enough initial data for the recommender system (to prevent overfitting), ensuring that items can be accessed outside the recommender system (to prevent a feedback loop between accessed items and recommended items), and encouraging participants to provide feedback on every narrative they interact with (to prevent participants from only providing feedback when they have strong opinions).

## Introduction

### Background

Recommender systems create personalized recommendations within a specific domain, suggesting items that may be of use to a user and helping quickly narrow down a potentially overwhelming number of options [1]. Recommender systems are used on global platforms such as Netflix—a movie streaming service—which uses other people’s movie ratings to recommend movies, Amazon—an e-commerce company—which uses frequently-bought-together items to recommend purchases, and Pandora—a music streaming service—which uses 450 musical attributes to recommend songs [2].

A range of health care applications for recommender systems have been examined, including the use of recommender systems to suggest prompts for counselors in a suicide prevention helpline chat [3], tailor care preference assessments in nursing homes [4], and identify expert physicians for specific diseases [5].

In this paper, we present an evaluation of NarraGive, the first recommender system for providing web-based recommendations from a collection of mental health recovery narratives.

### Lived Experience Narratives

Mental health recovery narratives are a subset of lived experience narratives, which are representations of a person’s experiences of physical or mental health and how that person has lived through and responded to those experiences [6]. The uses of lived experience narratives in health research have been extensively studied but with little focus on *which* narratives people engage with.

Studies have explored the use of lived experiences to encourage people to seek and sustain treatment, such as using narratives to improve health care participation in patients with breast cancer [7], promote smoking cessation in the African American community [8], and promote diabetes self-management [9] and diabetes medication adherence [10]. The use of lived experiences in support groups has also been studied, such as sharing stories in diabetes education in minority ethnic groups [11]. Some studies have provided medical students with narratives to facilitate learning and improve subsequent medical practices, such as using patient stories during practice placements [12] and learning about cancer pathology using narratives of patients who have experienced cancer [13].

Other studies have explored the use of lived experiences as a therapeutic tool for individuals, such as student nurses creating digital stories to challenge the “reality shock” of beginning clinical practice [14], young women telling their stories to reduce stress [15], women with eating disorders accessing recovery stories [16], service users with psychosis watching lived experience videos [17], incarcerated women telling their stories [18], patients with dementia using storytelling as a therapeutic tool [19], adults with diabetes engaging in lived experience support groups to reduce diabetes-related distress [20], painting trees to symbolize periods of one’s life as a starting point for telling a life story to treat depression and anxiety [21], and young people watching digital stories to reduce the prevalence of binge drinking [22].

Lived experience narratives have the potential to be used for a wide variety of purposes and, as a result—as documented previously—are frequently used in interventions. However, so far, the focus of health lived experience–based interventions has been solely on examining the effects of engaging with these narratives, with less focus on which *specific* narratives the participants are exposed to (though a few studies have placed emphasis on providing representative narratives [23] or particularly engaging and high-quality narratives [8]). Thus, while there have been studies evaluating the use of recommender systems in health care settings and, separately, evaluating the use of lived experience narratives, there have not been any lived experience narrative recommender systems developed before this study.

### The Problem Being Addressed

This is the first evaluation of a lived experience narrative recommender system. The design of such a recommender system has distinct challenges. For example, narratives are sensitive types of data that impose ethical requirements to protect both the narrator and the recipient. Therefore, the use of recommender systems needs to be informed by considerations about the curation and use of narratives [24-26]. The goal of our evaluation was to develop preliminary evidence to inform the future use and evaluation of recommender systems with lived experience narratives.

### The Narrative Experiences Online Intervention

#### Overview

The Narrative Experiences Online (NEON) study [27,28] evaluated whether having web-based access to people’s real-life stories of recovery from mental illness can be helpful for people who are experiencing psychosis or other mental health problems. This builds on the evidence base that indicates that receiving recovery narratives can support mental health [27]. In the NEON intervention, participants interact with a web application through which they can access a web-based collection of mental health recovery narratives (henceforth, narratives)—the NEON Collection.

#### Narrative Characterization

The development of the NEON Collection, including the narrative inclusion criteria, has been reported elsewhere [29]. In brief, recorded recovery narratives were obtained, always with consent, from existing collections and individual donations to the study. Only narratives that could be presented on the web in a single electronic file (eg, PDF, JPEG, and WAV) were included. Within these files, narratives were presented in a range of forms, including prose, poetry, audio recordings, video recordings, individual images, and sequential art. Each was presented by a single narrator only—there were no composite narratives. The narratives were deliberately chosen to be diverse [30]. All narratives in the NEON Collection were characterized using the Inventory of Characteristics of Recovery Stories (INCRESE) [31] to capture 77 different features of the narratives related to narrator characteristics, narrative content, and turning points. While we used selected INCRESE characteristics in our recommender system, the greater breadth of characteristics collected will support future secondary analyses. The trials opened with 348 narratives and closed with 659 narratives available.

#### Narrative Request Routes

There are 6 ways for participants to request narratives through the NEON intervention, which are internally documented as 1 of 8 request methods.

Textbox 1 summarizes the external and internal narrative request routes.

The NEON intervention home page has buttons corresponding to 4 of the 6 external narrative request routes: “Match me to a story (recommended),” “Get me a random story,” “Browse stories,” and “My stories.”

The first option uses NarraGive to recommend a single narrative that the participant has not seen before. NarraGive is a hybrid recommender system (meaning that it uses a combination of recommendation strategies [32]) that uses both content-based filtering (recommending narratives based on their content) and collaborative filtering (recommending narratives based on how other participants have rated them) to recommend narratives to participants.

The second option presents a randomly selected narrative that the participant has not seen before.

The third option allows participants to browse narratives grouped into categories based on the narratives’ INCRESE characteristics (Figures S1 and S2 in Multimedia Appendix 1)—some categories are based on the value of a single characteristic (eg, the narrator’s gender is “female”), and some are based on the value of multiple characteristics (eg, a positive narrative, defined as having an “upbeat” tone and an “escape” or “enlightenment” genre; Table S1 in Multimedia Appendix 1). Not all narratives are accessible through the category option.

The fourth option allows participants to access narratives that they have previously bookmarked or rated highly.

In addition, the internal request routes include whether NarraGive produced the recommendation using content-based filtering or collaborative filtering and whether a narrative selected from the “My stories” page was previously rated highly for hopefulness or manually bookmarked by the participant. One important benefit of having different narrative request routes is to prevent exposure bias, a well-known issue in recommender systems where participants are only presented with a subset of the available items, so they only provide ratings for that subset, with recommender systems unable to distinguish between disliked and unrated items and unknown and unrated items [33]. For example, the “Get me a random story” button might allow participants to access narratives that they would not otherwise be exposed to but that nonetheless may be beneficial.

**Narrative request mechanisms that participants use to access narratives (external routes) and the corresponding logs made by the intervention (internal routes).**

**External and internal narrative request routes**

- Participant clicks on the “Match me to a story (recommended)” button
  - Participant accesses a narrative recommended via content-based filtering.
  - Participant accesses a narrative recommended via collaborative filtering.
- Participant clicks on the “Get me a random story” button
  - Participant requests a random narrative.
- Participant clicks on the “Browse stories” button and selects a narrative
  - Participant makes a category-based request for a narrative.
- Participant clicks on the “My stories” button and selects a narrative
  - Participant requests a narrative that they have rated as hopeful.
  - Participant requests a narrative that they have marked as a favorite.
- Participant uses the intervention for the first time and is presented with their first narrative
  - Participant accesses their “first” narrative.
- Participant clicks on a narrative from a Narrative Experiences Online (NEON) communication
  - Participant accesses the suggested narrative in a reminder message aimed at prompting them to use the NEON intervention.

#### Narrative Feedback

After a participant has accessed a narrative through any request route, they are presented with 5 feedback questions (Table 1), and their responses to these questions are time-stamped and logged. The focus (hope, similarity, learning, and empathy) is based on the NEON Impact Model [29] developed through a systematic review [34] and qualitative [35] and experimental studies [36]. The measurement approach has been previously validated [29]. To maximize response rates, the first question is marked as compulsory. The other 4 questions are marked as optional, and the participant has the choice to answer either all or none of the optional questions. A set of 5 response values (for the 1 compulsory and 4 optional questions) forms a single rating, as does a single response value for the compulsory question. Ratings with optional questions answered are also referred to as optional ratings. Table 1 shows the questions, answer options, and numerical ranges (not visible to participants) of the questions and whether they are mandatory.

If a narrative is rerated, this overrides the previous rating (but the time-stamped logs of previous ratings are not deleted).

One benefit of recommender systems *requiring* a rating for each narrative is that this helps minimize selection bias, which occurs when participants are allowed to choose whether to rate the items, leading to ratings that are typically biased toward higher or more homogeneous ratings [33,37]. Selection bias is a well-known problem in recommender systems relying on explicit data.

**Table 1:** Questions, answer options, numerical ranges, and mandatory nature of narrative response data.

Question	Answer options	Range	Mandatory
How hopeful did the story leave you feeling? (hopefulness)	“Less hopeful than before,” “no change,” “a bit more hopeful,” and “much more hopeful”	−1 to 2	Yes
How similar was the storyteller to you? (similarity to the narrator)	“Not at all,” “a bit,” “quite a lot,” and “very much”	0 to 3	No
How similar was the storyteller’s life to your life? (similarity to the narrative)	“Not at all,” “a bit,” “quite a lot,” and “very much”	0 to 3	No
How much did you learn from the story? (learning)	“Not at all,” “a bit,” “quite a lot,” and “very much”	0 to 3	No
How emotionally connected did you feel with the story? (empathy)	“Not at all,” “a bit,” “quite a lot,” and “very much”	0 to 3	No

#### The NarraGive Recommender System

NarraGive is a hybrid recommender system. It uses one content-based and 2 collaborative filtering algorithms to allow for comparison of performance of the 3 algorithms using 2 distinct approaches to inform this new field of lived experience narrative recommendation. NarraGive was assembled using the Simple Python Recommendation System Engine library (SurPRISE; version 1.1.1; Nicolas Hug) for Python (version 3.6 and above), integrating implementations of filtering algorithms provided in these libraries [38]. NarraGive does not recommend previously requested narratives, types of narratives that a user has previously blocked, or individual narratives that a user has blocked.

The content-based filtering algorithm is based on the SurPRISE implementation of the k-nearest neighbor (kNN) algorithm. Although kNN is traditionally used as a collaborative filtering algorithm, NarraGive used an adapted version to measure the similarity between narratives, in which it uses their INCRESE characteristics to cluster together narratives in “neighborhoods” and recommend to participants unseen narratives that are similar to their other highly rated narratives. Narrative similarity is assessed using selected INCRESE characteristics, consisting of the INCRESE sections on narrator characteristics, narrative characteristics, narrative content, and turning points.

The selected collaborative filtering algorithms are the SurPRISE implementations of the singular value decomposition (SVD) and, to support comparison, SVD++. A broad introduction to these 2 algorithms is provided in the work by Hug [39]. These aim to capture the latent factors that determine how much a participant likes a narrative. NarraGive ran these 2 algorithms and selected the narrative with the highest predicted rating. Thus, the 2 algorithms served as distinct subsystems, so this evaluation will analyze the 2 subsystems separately to compare them. For the purposes of collaborative filtering, similarity between users is assessed using the demographic items collected in a “personal profile” created at first use and containing items describing participant demographics and format preferences. Multimedia Appendix 2 provides details on all items in the profile.

When making a narrative recommendation, narrative feedback ratings are weighted (with a hopefulness rating twice as influential as each of the individual optional ratings) and combined. This was due to the underlying theory that we developed on narratives making an impact on recipients, which emphasized hope creation as the most critical mechanism. When a participant requests a narrative from NarraGive, it internally generates 1 list per algorithm of the 10 narratives with the highest predicted rating. It then presents the highest-scoring narrative of these 30 to the participant. The participant is not shown the predicted rating, other internally generated narratives, or which of the 2 filtering mechanisms was used to generate the recommendation.

### The NEON Trials

The NEON intervention has been evaluated in 3 pragmatic randomized controlled trials with different populations. The NEON trial (ISRCTN11152837; N=739) is a definitive trial for people with experience of psychosis. The NEON for other (eg, nonpsychosis) mental health problems (NEON-O) trial (ISRCTN63197153; N=1023) is a definitive trial for people experiencing any other type of mental health problem. The NEON-C trial (ISRCTN76355273; N=54) is a feasibility trial with people who informally care for people experiencing mental health problems, which is not within the scope of this study. The NEON intervention was identical in all 3 trials. A separate instance of NarraGive was used for each trial, and there was no pooling of narrative feedback or recommendations among the 3 trials.

### Aims and Objectives

The aim of this study was to analyze the 3 recommender system algorithms used in NarraGive to inform future interventions using recommender systems in this new field of lived experience narrative recommendations. An evaluation of the impact of the NEON intervention using NarraGive has been reported elsewhere [40]. This study did not aim to provide an indication of NarraGive’s viability but rather to inform the development of future lived experience narrative recommender systems and guide design choices on collaborative versus content-based filtering algorithms.

The objectives were as follows:

1. To describe participant characteristics and patterns of narrative requests and feedback.
2. To evaluate the algorithms used in NarraGive by comparing collaborative-based and content-based narrative recommendations to inform future implementation approaches.

Objective 1 was addressed using data from the intervention version of NarraGive, and objective 2 was addressed using data from the final evaluated version.

## Methods

### Overview

An evaluation of NarraGive was conducted using data from the NEON and NEON-O trials, structured using the framework for evaluating recommender systems (FEVR), which was developed through a review of recommender system evaluation work [41]. The FEVR defines a set of components intended to guide the design of a recommender system evaluation.

After the NEON trials closed, logging files describing interactions with trial procedures and the NEON intervention were downloaded for analysis. These files included trial allocation, baseline demographic characteristics, personal profiles, narrative characteristics, narratives that the participants requested and the corresponding internal narrative request route, and participants’ ratings. All log entries were time-stamped.

### Ethical Considerations

The NEON study trial protocol and an update have been published elsewhere [27,28]. Ethics approval was obtained in advance of trial start from a UK National Health Service Research Ethics Committee (Leicester Central Research Ethics Committee; 19/EM/0326). All participants provided web-based informed consent for the use of their data for research purposes, and all study data were pseudonymous, with each participant’s data linked by a unique ID. Some participants were compensated (£20 [US $25.59] vouchers) for some data collection rounds, as described in our trial protocol.

### Participants

The NEON trial included participants who (1) had experience of psychosis in the previous 5 years, (2) had experience of mental health–related distress in the previous 6 months, (3) resided in England, (4) were aged ≥18 years, (5) were capable of accessing or being supported to access the internet on a PC or mobile device or at a community venue, (6) were able to understand written and spoken English, and (7) were capable of providing web-based informed consent.

The NEON-O trial included participants who (1) had experience of mental health problems other than psychosis in the previous 5 years, (2) had experience of mental health–related distress in the previous 6 months, (3) resided in England, (4) were aged ≥18 years, (5) were capable of accessing or being supported to access the internet on a PC or mobile device or at a community venue, (6) were able to understand written and spoken English, and (7) were capable of providing web-based informed consent. It excluded participants eligible for the NEON trial.

Our study included participants from the NEON trials’ intention-to-treat samples [27].

### Sample Size

Both trials were powered on the mean item score for the 12 subjective items in the Manchester Short Assessment of Quality of Life (MANSA) as collected at baseline and the 52-week follow-up [42], and hence, the sample size was chosen on this basis.

For the NEON trial, a total sample size of 684 was chosen to provide 90% power to detect a minimal clinically important effect size (Cohen *d*) of 0.27 (SD 0.9 [43]; power=90%; *P*=.05), allowing for 20% attrition. The planned analyzable sample size was 546.

For the NEON-O trial, the SD of the MANSA scores for the study population was estimated from baseline data provided by the first 350 enrolled participants (see the study by Rennick-Egglestone et al [27] for the rationale). A total sample size of 994 was selected to provide 90% power to detect a minimal clinically important effect size (Cohen *d*) of 0.27 (SD 0.94; power=90%; *P*=.05), allowing for 40% attrition, which was estimated from the completion rates for interim data. The planned analyzable sample size was 596.

Both trials recruited their planned samples and were allowed to overrecruit (N=739 for the NEON trial and N=1023 for the NEON-O trial). The final attrition rates were 23.5% (NEON trial) and 44.8% (NEON-O trial).

### Evaluation Framework

Table 2 describes the FEVR components that were selected to define the evaluation.

**Table 2:** Framework for evaluating recommender systems (FEVR) components defining the NarraGive evaluation.

FEVR component			Brief description
**Evaluation objectives**			
	Overall goal	To evaluate whether the recommender system NarraGive supported participants in finding helpful narratives	
	Stakeholders	Participants in the NEON^a^ and NEON-O^b^ trials’ ITT^c^ samples	
	Properties	Prediction accuracy, usage prediction, diversity, coverage, and unfairness across participants	
**Evaluation principles**			
	Hypothesis or research question	Objective 1: To describe participant characteristics and patterns of narrative requests and feedback Objective 2: To evaluate the NarraGive recommender system by comparing collaborative-based and content-based narrative recommendations	
	Control variables	Randomized data set that is split 75:25 between the training set (to train the algorithms) and the testing set (to evaluate the metrics)	
	Generalization power	Use of real-world data from participants with mental health problems; limited due to variation in system use	
	Reliability	Cross-validation with repeated initialization of collaborative filtering algorithms	
Experiment type			Offline evaluation
**Evaluation aspects**			
	Types of data	Explicit ratings	
	Data collection	Participant ratings (prompted after every narrative access)	
	Data quality and biases	Platform bias from suggested narratives	
	Evaluation metrics	Normalized mean absolute error (for prediction accuracy) Mean average precision per participant (for usage prediction) Intralist diversity (for diversity) Item space coverage (for coverage) Overestimation of unfairness (for unfairness across participants)	
	Evaluation system	NEON intervention web application	

^a^NEON: Narrative Experiences Online.

^b^NEON-O trial: NEON for other [eg, nonpsychosis] mental health problems) trial.

^c^ITT: intention to treat.

### Recruitment

Participants were recruited across England from March 9, 2020 (both trials), to March 1, 2021 (NEON trial), or March 26, 2021 (NEON-O trial). The trials used a mixed web-based and offline approach to recruit participants. Recruitment was through paid web-based advertising on mental health websites; promotional messaging distributed by a range of community groups and health care practices; promotional messaging distributed on Facebook, Twitter (subsequently rebranded as X), and Google (with the reach of messages enhanced through payments); media appearances by the central study team; and the work of clinical research officers in 11 secondary care mental health trusts.

Clinical research officers approached participants in person and distributed promotional messaging through local authorized channels such as mailing lists of service users who had consented to be contacted about research studies. All promotional advertising and messaging conformed to principles approved in advance by the supervising research ethics committee [44].

### Registration

All recruitment approaches directed potential participants to a web-based eligibility checking interface that requested responses to a series of questions specified in the trial protocol. All responses were self-rated. No formal diagnosis of a mental health condition was required for participation. Trial allocation was determined through responses, and eligible potential participants were provided with access to a tailored web-based participant information sheet. Participants subsequently completed a web-based informed consent form by providing an email address and optional telephone number.

The consent process was concluded by clicking on a link in an auto-generated email to validate the email address. After confirming consent, participants completed web-based forms to collect baseline demographic and clinical data and were then randomized using a web-based system validated by a clinical trial unit to the intervention or control arm. Demographic items were age (in years), gender (female, male, or other), ethnicity, region of residence, highest educational qualification, lifetime use of primary care mental health services, lifetime use of specialist mental health services, current use of mental health services in relation to psychosis (NEON trial only), main mental health problem in the last month, best description of recovery status, residential status, and employment status.

Intervention arm users gained immediate access to the NEON intervention until trial end (September 22, 2022), whereas control arm users gained access after completing the 52-week follow-up questionnaires and until trial end. Data on NEON intervention use by both intervention and control group users are within the scope of this study.

### Analysis

#### Objective 1: Describe Participant Characteristics and Patterns of Narrative Requests and Feedback

##### Participant Characteristics

The demographic and clinical characteristics of participants randomized to each trial were described using means and SDs for normally distributed data and counts with percentages for categorical data. Descriptive statistics were calculated for all baseline demographic data items.

Following UK Data Service guidance on statistical disclosure [45], ethnicity responses were grouped into 2 categories (White British and other ethnicity) due to the small number of participants in most ethnicity categories, although recognizing that this could be perceived as a reductive approach to ethnicity data. “Current mental health problem” also comprised categories with low numbers of participants, so relevant rows were shown as “<5” with no percentage, and other rows were shown as “<10” with no percentage to avoid being able to infer other values.

##### Patterns of Narrative Requests and Feedback

Data on participant narrative requests and narrative feedback were taken from log files and used to calculate per-trial summary statistics for the number of participants, number of participants who requested at least one narrative, number of narratives at the start and end of the trial, number of narratives given at least one rating, number of narrative requests, number of narrative ratings, number of optional ratings, number of ratings per narrative, number of ratings per rated narrative, length of intervention use by participants, and narrative access routes.

While providing feedback on narratives was encouraged, it was possible for the participant to navigate away from the page and not submit any feedback; therefore, the number of narrative ratings may be smaller than the number of narrative requests, so these figures were reported separately.

Statistics for the number of ratings per narrative present 2 sets of figures with different selection criteria: those including only data for narratives that received at least one rating and those including data for all narratives. This breakdown shows how many ratings NarraGive had access to as it could only access rated narratives.

Nonparametric data were presented as medians and IQRs. Category data were presented as counts with percentages.

#### Objective 2: Evaluate the NarraGive Recommender System by Comparing Collaborative-Based and Content-Based Narrative Recommendations

##### Overview

The 3 algorithms (kNN, SVD, and SVD++) were trained and tested using all the available data, representing the point in time at which the trials closed. Training an algorithm involves providing it with a set of data that it can use to create predictions for missing data points. Testing an algorithm involves obtaining these predictions and measuring a feature of those predictions.

The results for objective 2 were obtained using the SurPRISE library (version 1.1.3) for Python (version 3.10.7). Only participants who provided at least one rating and narratives that were given at least one rating were included (as SurPRISE uses participant-item rating pairs as the basis for its predictions), which mirrors the information that NarraGive had access to during the intervention.

This study evaluated NarraGive using the metrics outlined in Textbox 2, applied separately to the content-based algorithm (kNN) and the collaborative filtering–based algorithms (SVD and SVD++).

There are 2 types of metrics: metrics that compare predicted ratings with actual ratings (prediction-based metrics) and metrics that measure a feature of the top-n predicted items (feature-based metrics). Prediction-based metrics include prediction accuracy, usage prediction, and unfairness across participants. Feature-based metrics include diversity and coverage. For prediction-based metrics, there is no standard data-splitting strategy [46], so the data set is split into a training set (75%) and a testing set (25%). For feature-based metrics, the entire data set is used as the training set.

NarraGive only used the first 3 sets of ratings (hopefulness, similarity to the narrator, and similarity to the narrative) to inform its recommendations as these 3 questions had been validated in a feasibility study [29] and the remaining 2 questions were added after the feasibility study. Therefore, only the first 3 sets of ratings were used in the evaluation.

The hopefulness ratings were normalized, which in this case involved shifting the ratings to use the same rating scale as that of the 4 optional questions.

The evaluated version of NarraGive presented in this paper used the same training data as the intervention version of NarraGive with 3 minor modifications. First, where the narratives’ INCRESE characteristics were updated during the trials (eg, to correct human error in inputting characteristics), this evaluation only used the final set of uploaded characteristics. Second, during the intervention, NarraGive filtered out previously requested and blocked narratives. This evaluation included these narratives as the predictions themselves were not influenced by whether a narrative was blocked or previously requested (ie, blocked and previously requested narratives were filtered out after the prediction process in the trial implementation), which could affect, for example, coverage metrics. Third, during the NEON trials, some accounts were removed due to suspected repeat registrations [27]; this evaluation removed all ratings from those participants even though NarraGive may have initially used those ratings.

The logs that were recorded during the intervention did not include NarraGive’s internal recommendation lists and instead only recorded the single narrative that was selected to show to the participants. Therefore, using the intervention version of NarraGive would have prevented any comparison of its subsystems and would have allowed for only a limited analysis of its performance as a whole.

The results from objective 1 (about participants and their use of the system) used the data collected from the live intervention, whereas the results from objective 2 (about NarraGive and its subsystems) used the evaluation version of NarraGive.

During a previous feasibility study of NEON (N=25 mental health service users), 465 ratings were collected for the initial set of narratives in the NEON Collection [29]. NarraGive had access to these ratings in the NEON and NEON-O trials to reduce the “cold start” problem, where recommender systems perform poorly for new items and participants [1]. The evaluation excluded these ratings to ensure that NarraGive was only evaluated on data collected live during the NEON intervention.

The SVD and SVD++ algorithms were both randomly initialized according to a normal distribution [47], and the 75:25 split between training and testing sets was also random and calculated using NumPy (a package for scientific computing with Python) [48], where “fresh, unpredictable entropy will be pulled from the OS” [48]. To account for the randomness, cross-validation was performed. The data set was split into 4 folds, with a different fold used as the testing set each time, and the SVD and SVD++ algorithms were reinitialized each time. Medians and IQRs were reported.

An additional exploratory analysis was conducted to determine how the accuracy changed over time. For each month between June 2020 and July 2022 inclusive, data up to but not including the first day of each month were used for training and testing, and the accuracy was measured (using the same accuracy metric as for the main NarraGive evaluation).

**The 5 metrics for evaluating NarraGive.**

**Metric and metric category**

- Prediction accuracy
  - Normalized mean absolute error
- Usage prediction
  - Mean average precision per participant
- Diversity
  - Intralist diversity
- Coverage
  - Item space coverage
- Unfairness across participants
  - Overestimation of unfairness

##### Prediction Accuracy

Prediction accuracy is the extent to which a recommender system can predict participant ratings [41]. The root-mean-square error (RMSE) and mean absolute error (MAE) [49] are 2 of the most commonly used metrics for evaluating rating prediction accuracy. The MAE uses the absolute difference between the predicted and true ratings, whereas the RMSE squares this difference, which results in the RMSE penalizing inaccurate predictions more [1].

The intervention was designed to be used over time rather than as a one-off, so the accuracy metric should primarily capture the *overall* accuracy rather than emphasizing occasional large inaccuracies (ie, an inaccurate prediction off by 2 points followed by a completely accurate prediction should be treated as no worse than 2 inaccurate predictions off by 1 point each), and this is better achieved using the MAE. Because the hopefulness ratings were normalized, the prediction accuracy metric was the normalized MAE (NMAE).

Different variations in the MAE have been reported in the literature. In particular, some versions square root the averaged summation [1], whereas others do not [47,50]. This evaluation uses SurPRISE’s in-built MAE calculation, which does not use a square root.

A lower NMAE indicates greater prediction accuracy. For NarraGive, the scale ranges from 0 (greatest prediction accuracy) to 4 (equation 1 in Multimedia Appendix 3).

##### Usage prediction

Usage prediction is the rate of correct recommendations in a setting where recommendations are classified as 1 of 2 options: relevant or nonrelevant [41]. An item is relevant to a participant when the participant’s rating for it meets a predefined numerical threshold (where the threshold is participant independent and defined per question).

There are 2 common metrics for measuring usage prediction: precision and recall. Precision measures how likely it is that a recommended item is relevant and is defined as the ratio of relevant selected items to the total number of selected items [49]. Recall, conversely, measures how likely it is that a relevant item is selected and is defined as the ratio of relevant selected items to the total number of relevant items [49].

As the length of the recommendation list increases, recall improves, whereas precision worsens [1,49]. The length of NarraGive’s internal recommendation list is 10, which is relatively short (compared to, for example, a search engine that recommends tens or hundreds of web pages), meaning that it is impossible to achieve a meaningfully high recall score, so the metric for usage prediction was precision.

As usage prediction is usually used for measuring how relevant a *list* of recommendations is, this evaluation used NarraGive’s internal recommendation list (consisting of a 10-narrative list produced using content-based filtering and two 10-narrative lists produced using collaborative filtering). As the participants do not see this list, only metrics that focus on the characteristics of the list as a whole—rather than focusing on the order *within* the list—were used (ie, where the list is treated more like a mathematical set than an ordered list as the ordering beyond the first item does not affect participants), and metrics that exclusively evaluate ranking order were not used.

The analysis of recommender system evaluations by Herlocker et al [49] showed that accuracy metrics can be divided into equivalence classes. One of these classes comprises all metrics that are averaged overall, and one of these classes comprises per-user correlation metrics and the mean average precision per-user metric. To ensure that this analysis of NarraGive captured its performance as widely as possible, a variation of precision that falls into a different equivalence class from that of the NMAE was used, namely, the mean average precision per participant (hereafter, precision).

As the ratings are on a 4-point scale, they need to be converted to a binary scale that classifies recommendations as either relevant or nonrelevant. For optional questions, relevance was defined as “a bit,” “quite a lot,” or “very much.” For hopefulness, relevance was defined as “no change,” “a bit more hopeful,” or “much more hopeful.”

Higher precision indicates a greater proportion of relevant narratives. The scale ranges from 0 (least precision) to 1 (equation 2 in Multimedia Appendix 3).

##### Diversity

Diversity measures how varied the recommended items are [41]. The current metrics for diversity [41,50] are intralist diversity (ILD) and variations thereof. ILD was developed by Ziegler et al [51], and variations include the rank-sensitive ILD metric by Vargas and Castells [52]. Similar to usage prediction, because the lists used to calculate diversity came from NarraGive’s internal recommendation list and the ILD by Ziegler et al [51] is permutation insensitive (ie, the position of recommendations on the list does not affect the diversity score), this metric was used, with cosine similarity as the distance metric calculated using the narratives’ INCRESE characteristics.

The original study defined ILD on a per-list basis (ie, for the recommendation list of one participant). This metric has been expanded in this study to be averaged over all participants’ lists to produce an overall ILD value.

The lower the ILD value, the greater the diversity among the recommended items. The scale ranges from −1 (most diverse) to 1 (equation 3 in Multimedia Appendix 3).

##### Coverage

Coverage can be split into participant space coverage and item space coverage [41]. Participant space coverage is the proportion of participants who can be provided with recommendations by the recommender system [1]. The threshold for being provided recommendations is low—a participant needs to have rated at least one narrative (which is achieved when they first access the intervention as it is compulsory to provide a response for the first narrative); thus, participant space coverage was not used. A variation of participant space coverage assesses the proportion of participants that can be recommended *high-quality* items (ie, items with a predicted rating above a predefined threshold). This notion of variable quality among participants is addressed more thoroughly using an *unfairness across participants* metric instead.

Item space coverage is the proportion of items that the recommender system can recommend [1]. Ge et al [53] further split item space coverage into prediction coverage and catalog coverage. They defined prediction coverage as the proportion of items for which the recommender system can produce a predicted rating and catalog coverage as the proportion of items that are recommended in a series of recommendation lists. Because there is no predefined limit to when NarraGive can produce a predicted rating for a narrative, prediction coverage was used.

The definition of catalog coverage by Ge et al [53] captures the set of recommended items produced over time for a single participant (ie, the items that would have been recommended to the participant if they had asked for recommendations at that time; this is different from the set of recommended items that the participant requested and was actually presented with over time).

To capture the *overall* coverage, the proportion of narratives that are recommendable is measured, where a narrative is recommendable if, for at least one participant, the narrative appears in NarraGive’s internal recommendation list.

Other versions of coverage use only the top recommendation, but as there are more narratives than there are participants, this would upper bound the item space coverage at approximately three-quarters for the NEON trial—total number of recommendations (which is equal to the number of participants who rated at least one narrative as there is 1 recommendation per participant) divided by the number of narratives that were rated at least once. For longer recommendation lists (such as 10), because recommender system algorithms cannot always produce a predicted rating for each item, a participant’s list may be less than the desired length. For this evaluation, a length of 10 was sufficient to ensure that the total number of recommendations being considered across all participants was greater than the number of narratives.

A higher item space coverage value indicates greater item coverage. The scale ranges from 0 (lowest item coverage) to 1 (equation 4 in Multimedia Appendix 3).

##### Unfairness Across Participants

Unfairness across participants measures whether participants are treated fairly either at the group level (participants in the same group are treated fairly) or at the individual level (participants who are similar are treated fairly) [41].

NarraGive is designed for use in a health care setting—a setting in which protected characteristics such as disability are critical to attend to. It would be crude to stipulate that, for example, all participants should have an equal probability of being recommended a narrative about wheelchair users as this would be far more relevant to some participants than others (and, indeed, a recommender system’s entire purpose is to provide *personalized* rather than generic recommendations). As acknowledged by Yao and Huang [54], “in tasks such as recommendation, user preferences are indeed influenced by sensitive features such as gender, race, and age. Therefore, enforcing demographic parity may significantly damage the quality of recommendations.”

Thus, they proposed 4 metrics: value unfairness, absolute unfairness, underestimation of unfairness, and overestimation of unfairness. Value unfairness “occurs when one class of user is consistently given higher or lower predictions than their true preferences.” Absolute unfairness “measures inconsistency in absolute estimation error across user types.” Underestimation of unfairness “measures inconsistency in how much the predictions underestimate the true ratings.” Overestimation of unfairness “measures inconsistency in how much the predictions overestimate the true ratings.”

NarraGive is implemented in a health care context in which the principle of harm avoidance is crucial. Therefore, one of the most important factors to consider is whether NarraGive is recommending potentially harmful narratives to participants. The metric used to measure this aspect is the overestimation of unfairness.

Overestimation of unfairness measures how much NarraGive consistently overestimates the predicted rating of narratives (ie, how often a participant rates a narrative lower than NarraGive expected) within a disadvantaged subset of the participants and compares this to the overestimation in the nondisadvantaged group.

Participants were divided into groups based on their demographic characteristics. The first grouping was by ethnicity as having a minority ethnicity predicts mental health problems [55], and the second grouping was by gender, informed by Sex and Gender Equity in Research guidelines [56].

The disadvantaged group for the gender comparison was defined as either “Female” or “Other.” The disadvantaged group for the ethnicity comparison was defined as “Irish,” “Gypsy or Irish Traveller,” “Any other White background,” “White and Black Caribbean,” “White and Black African,” “White and Asian,” “Any other Mixed/Multiple ethnic background,” “Indian,” “Pakistani,” “Bangladeshi,” “Chinese,” “Any other Asian background,” “African,” “Caribbean,” “Any other Black/African/Caribbean background,” “Arab,” and “Any other ethnic group.”

The baseline demographic information was used for measuring unfairness between participants as the questions were compulsory, so there was higher completeness of the baseline data than of the personal profile as well as greater granularity with the range of possible answers. The overestimation of unfairness is defined according to the study by Yao and Huang [54].

A lower overestimation of unfairness value indicates that there is less disparity between overestimation among disadvantaged participants and among nondisadvantaged participants. The scale ranges from 0 (least unfair) to 4 (equation 5 in Multimedia Appendix 3).

##### Other Categories

Zangerle and Bauer [41] detailed 10 categories of evaluation metrics that can be used in the FEVR. Of these, 5 (discussed previously) were used in evaluating NarraGive, and the other 5—ranking, novelty, serendipity, fairness across items, and business oriented—were not used for the reasons described in Multimedia Appendix 4 [33,41,57].

## Results

### Objective 1: Describe Participant Characteristics and Patterns of Narrative Requests and Feedback

#### Participant Characteristics

The baseline sociodemographic and clinical characteristics of participants in the NEON (N=739) and NEON-O (N=1023) trials are shown in Table 3.

An exploration of the baseline differences has been reported elsewhere [58].

**Table 3:** Baseline sociodemographic and clinical characteristics of Narrative Experiences Online (NEON) and NEON for other (eg, nonpsychosis) mental health problems trial (NEON-O) participants.

			NEON baseline (N=739)		NEON-O baseline (N=1023)
**Gender, n (%)**					
	Female	443 (59.9)		811 (79.3)	
	Male	274 (37.1)		184 (18)	
	Other	16 (2.2)		18 (1.8)	
Age (years), mean (SD)			34.8 (12)		38.4 (13.6)
**Ethnicity, n (%)**					
	White British	561 (75.9)		827 (80.8)	
	Other ethnicity	172 (23.3)		185 (18.1)	
**Region of residence, n (%)**					
	East of England	53 (7.2)		61 (6)	
	London	166 (22.5)		210 (20.5)	
	Midlands	112 (15.2)		203 (19.8)	
	North East and Yorkshire	80 (10.8)		102 (10)	
	North West	66 (8.9)		98 (9.6)	
	South East	133 (18)		214 (20.9)	
	South West	123 (16.6)		125 (12.2)	
**Highest educational qualification, n (%)**					
	No qualification	51 (6.9)		30 (2.9)	
	O-levels or GCSE^a^	117 (15.8)		116 (11.3)	
	A-levels or AS^b^-levels or NVQ^c^ or equivalent	278 (37.6)		327 (32)	
	Degree-level qualification	207 (28)		349 (34.1)	
	Higher degree-level qualification	80 (10.8)		191 (18.7)	
**Living arrangement, n (%)**					
	Alone	215 (29.1)		229 (22.4)	
	With others	524 (70.9)		794 (77.6)	
**Employment status, n (%)**					
	Employed	277 (37.5)		586 (57.3)	
	Sheltered employment	10 (1.4)		6 (0.6)	
	Training and education	76 (10.3)		106 (10.4)	
	Unemployed	356 (48.2)		272 (26.6)	
	Retired	20 (2.7)		53 (5.2)	
**Current mental health problem, n (%)**					
	I don’t want to say	20 (2.7)		14 (1.4)	
	I did not experience mental health problems	19 (2.6)		31 (3)	
	Developmental disorder such as learning disability	15 (2)		12 (1.2)	
	Eating disorder	15 (2)		45 (4.4)	
	Mood disorder	265 (35.9)		626 (61.2)	
	Personality disorder	138 (18.7)		123 (12)	
	Schizophrenia or other psychosis	154 (20.8)		<5 (<1)	
	Stress-related disorder	82 (11.1)		152 (14.9)	
	Substance-related disorder	25 (3.4)		<10 (<1)	
**Lifetime user of primary care mental health services, n (%)**					
	Yes	698 (94.5)		949 (92.8)	
	No	35 (4.7)		64 (6.3)	
**Current use of mental health services for psychosis, n (%)**					
	No contact with any NHS^d^ service	100 (13.5)		N/A^e^	
	General practitioner	234 (31.7)		N/A	
	Primary care counselor	59 (8)		N/A	
	IAPT^f^	56 (7.6)		N/A	
	Specialist community mental health team	261 (35.3)		N/A	
	Mental health inpatient in hospital	18 (2.4)		N/A	
**How would you best describe your recovery?, n (%)**					
	I don’t want to say	48 (6.5)		64 (6.3)	
	Not yet thinking about recovery	91 (12.3)		64 (6.3)	
	Working on recovery	510 (69)		784 (76.6)	
	Living beyond disability	84 (11.4)		101 (9.9)	

^a^GCSE: General Certificate of Secondary Education.

^b^AS: Advanced Subsidiary.

^c^NVQ: National Vocational Qualification.

^d^NHS: National Health Service.

^e^N/A: not applicable; indicates a question that participants were not asked; in particular, only NEON trial participants were asked about their current use of mental health services.

^f^IAPT: Improving Access to Psychological Therapies.

#### Patterns of Narrative Requests and Feedback

Table 4 shows summary statistics on the participants, narratives, narrative requests, narrative ratings, intervention use length, and narrative request routes.

A histogram of the lengths of intervention use is shown in Figure S1 in Multimedia Appendix 5. In the NEON trial, 12.4% (92/739) of the participants used the intervention only once, whereas in the NEON-O trial, 19.45% (199/1023) of the participants used the intervention only once. Lengths of >400 days were merged to prevent participant identifiability. The lengths of intervention use for the first 30 days are shown in Figure S2 in Multimedia Appendix 5, with participants who only used the intervention once (“single-use participants”) removed to show only nonzero time lengths.

Tables 5 and 6 show the number of narrative rating values that each question received for ratings from NEON trial participants.

Tables 7 and 8 show the number of narrative rating values that each question received for ratings from NEON-O trial participants.

**Table 4:** Number of narrative requests via the content-based filtering internal access route, collaborative filtering internal access route, and all other internal access routes.

		NEON^a^	NEON-O^b^
**Participants, n (%)**		739 (100)	1023 (100)
	Participants who requested at least one narrative	365 (49.4)	562 (54.9)
	Participants who requested and rated at least one narrative	284 (38.4)	409 (40)
Narratives at the start of the trial, n (%)		348 (100)	348 (100)
**Narratives at the end of the trial, n (%)**		657 (100)	657 (100)
	Narratives given at least one rating	375 (57.1)	366 (55.7)
Narrative requests, n (%)		3762 (100)	3548 (100)
**Narrative ratings, n (%)**		2288 (100)	1896 (100)
	Optional ratings	538 (23.5)	538 (28.4)
Ratings per narrative, median (IQR)		1 (0-4)	1 (0-3)
Ratings per rated narrative, median (IQR)		3 (2-6)	2 (1-5)
Length of intervention use, median (IQR)		20 days, 22 hours, and 17 minutes (0 days, 0 minutes, and 0 minutes-251 days, 0 minutes, and 0 minutes)	0 days, 0 hours, and 16 minutes (0 days, 0 hours, and 0 minutes-59 days, 19 hours, and 7 minutes)
Content-based filtering narrative request route, n (%)		554 (14.7)	554 (15.6)
Collaborative filtering narrative request route, n (%)		1113 (29.6)	763 (21.5)
Other narrative request route, n (%)		2095 (55.7)	2232 (62.9)

^a^NEON: Narrative Experiences Online.

^b^NEON-O: NEON for other (eg, nonpsychosis) mental health problems trial.

**Table 5:** Distribution of narrative rating values for the Narrative Experiences Online trial participants.

	−1, n (%)	0, n (%)	1, n (%)	2, n (%)
Hopefulness ratings (N=2288)	202 (8.83)	901 (39.38)	838 (36.63)	347 (15.17)

**Table 6:** Distribution of narrative rating values for the Narrative Experiences Online trial participants (N=538 ratings).

	0, n (%)	1, n (%)	2, n (%)	3, n (%)
Similarity to the narrator ratings	132 (24.5)	196 (36.4)	152 (28.3)	58 (10.8)
Similarity to the narrative ratings	103 (19.1)	144 (26.8)	173 (32.2)	118 (21.9)
Learning ratings	104 (19.3)	193 (35.9)	181 (33.6)	60 (11.2)
Empathy ratings	155 (28.8)	206 (38.3)	126 (23.4)	51 (9.5)

**Table 7:** Distribution of narrative rating values from Narrative Experiences Online for other (eg, nonpsychosis) mental health problems trial participants (N=1896 narrative ratings).

	−1, n (%)	0, n (%)	1, n (%)	2, n (%)
Hopefulness ratings (N=1896 narrative ratings)	206 (10.86)	845 (44.57)	649 (34.23)	196 (10.34)

**Table 8:** Distribution of narrative rating values from Narrative Experiences Online for other (eg, nonpsychosis) mental health problems trial participants (N=538 ratings).

	0, n (%)	1, n (%)	2, n (%)	3, n (%)
Similarity to the narrator ratings	195 (36.2)	211 (39.2)	104 (19.3)	28 (5.2)
Similarity to the narrative ratings	145 (27)	157 (29.2)	168 (31.2)	68 (12.6)
Learning ratings	134 (24.9)	242 (45)	144 (26.8)	18 (3.3)
Empathy ratings	236 (43.9)	193 (35.9)	90 (16.7)	19 (3.5)

### Objective 2: Evaluate the NarraGive Recommender System

#### Overview

The best results (per metric per trial) are italicized. Where 2 values are equal, neither was better than the other.

For rating sets, better means that all 6 values (across both trials) were better than the 2 corresponding values for the other 2 rating sets, with *N/A* if no rating set was better.

For algorithms, we identified the filtering approach that was better (if any), comparing the content-based and collaborative subsystems of NarraGive per rating set across both trials. Specifically, if the kNN value was better than both SVD and SVD++ values, then we identified content-based filtering as better. If both SVD and SVD++ values were better than the kNN value, then we identified collaborative filtering as better. If neither the kNN nor SVD and SVD++ was better than the other, then the value was calculated per trial.

For trials, better means that each of the 9 values was better than the corresponding value in the other trial, with *N/A* if neither trial was better.

#### Prediction Accuracy

Tables 9 and 10 show the NMAE of the kNN, SVD, and SVD++ algorithms when trained and tested on the hopefulness, similarity to the narrator, and similarity to the narrative ratings using NEON and NEON-O trial data, respectively.

For NMAE, better means lower.

Hopefulness was the better rating set, collaborative filtering was the better approach, and NEON-O was the better trial.

**Table 9:** Normalized mean average error (NMAE; using Narrative Experiences Online [NEON] trial data).

NMAE (NEON trial)	Hopefulness, median (IQR)	Similarity to the narrator, median (IQR)	Similarity to the narrative, median (IQR)
kNN^a^	0.686 (0.670-0.703)	1.070 (1.059-1.077)	1.150 (1.140-1.153)
SVD^b^	0.650 (0.638-0.664)	1.043 (1.035-1.047)	1.098 (1.076-1.121)
SVD++	*0.646 (0.639-0.654)* ^c^	1.044 (1.038-1.049)	1.099 (1.080-1.120)

^a^kNN: k-nearest neighbor.

^b^SVD: singular value decomposition.

^c^Best result is italicized (per metric per trial).

**Table 10:** Normalized mean average error (NMAE; using Narrative Experiences Online for other [eg, nonpsychosis] mental health problems trial [NEON-O] data).

NMAE (NEON-O trial)	Hopefulness, median (IQR)	Similarity to the narrator, median (IQR)	Similarity to the narrative, median (IQR)
kNN^a^	0.685 (0.677-0.697)	0.998 (0.992-1.006)	1.099 (1.097-1.106)
SVD^b^	0.650 (0.641-0.659)	0.978 (0.972-0.986)	1.076 (1.066-1.093)
SVD++	*0.644 (0.635-0.655)* ^c^	0.983 (0.977-0.989)	1.079 (1.065-1.099)

^a^kNN: k-nearest neighbor.

^b^SVD: singular value decomposition.

^c^Best result is italicized (per metric per trial).

#### Usage prediction

Tables 11 and 12 show the precision of the kNN, SVD, and SVD++ algorithms when trained and tested on the hopefulness, similarity to the narrator, and similarity to the narrative ratings using NEON and NEON-O trial data, respectively.

For precision, better means higher.

Hopefulness was the better rating set, there was no better filtering approach, and NEON was the better trial.

**Table 11:** Precision (using Narrative Experiences Online [NEON] trial data).

Precision (NEON trial)	Hopefulness, median (IQR)	Similarity to the narrator, median (IQR)	Similarity to the narrative, median (IQR)
kNN^a^	0.255 (0.250-0.258)	0.054 (0.052-0.055)	0.057 (0.056-0.060)
SVD^b^	*0.256 (0.250-0.259)* ^c^	0.053 (0.052-0.055)	0.057 (0.055-0.060)
SVD++	*0.256 (0.250-0.259)*	0.053 (0.051-0.055)	0.057 (0.055-0.060)

^a^kNN: k-nearest neighbor.

^b^SVD: singular value decomposition.

^c^Best result is italicized (per metric per trial).

**Table 12:** Precision (using Narrative Experiences Online for other [eg, nonpsychosis] mental health problems trial [NEON-O] trial data).

Precision (NEON-O trial)	Hopefulness, median (IQR)	Similarity to the narrator, median (IQR)	Similarity to the narrative, median (IQR)
kNN^a^	*0.181 (0.172-0.192)* ^b^	0.037 (0.034-0.040)	0.041 (0.038-0.043)
SVD^c^	0.180 (0.172-0.191)	0.037 (0.035-0.039)	0.041 (0.039-0.043)
SVD++	0.180 (0.172-0.192)	0.036 (0.033-0.039)	0.040 (0.038-0.043)

^a^kNN: k-nearest neighbor.

^b^Best result is italicized (per metric per trial).

^c^SVD: singular value decomposition.

#### Diversity

Tables 13 and 14 show the ILD of the kNN, SVD, and SVD++ algorithms when trained and tested on the hopefulness, similarity to the narrator, and similarity to the narrative ratings using NEON and NEON-O trial data, respectively.

For ILD, better means lower.

There was no better rating set, collaborative filtering was the better approach for the NEON trial, there was no better approach for the NEON-O trial, and NEON-O was the better trial.

**Table 13:** Intralist diversity (ILD; using Narrative Experiences Online [NEON] trial data).

ILD (NEON trial)	Hopefulness, median (IQR)	Similarity to the narrator, median (IQR)	Similarity to the narrative, median (IQR)
kNN^a^	0.542 (0.542-0.542)	0.540 (0.540-0.540)	0.538 (0.538-0.538)
SVD^b^	0.531 (0.530-0.532)	0.539 (0.538-0.539)	0.538 (0.537-0.538)
SVD++	*0.530 (0.530-0.531)* ^c^	0.539 (0.538-0.539)	0.538 (0.538-0.539)

^a^kNN: k-nearest neighbor.

^b^SVD: singular value decomposition.

^c^Best result is italicized (per metric per trial).

**Table 14:** Intralist diversity (ILD; using Narrative Experiences Online for other [eg, nonpsychosis] mental health problems trial [NEON-O] trial data).

ILD (NEON-O trial)	Hopefulness, median (IQR)	Similarity to the narrator, median (IQR)	Similarity to the narrative, median (IQR)
kNN^a^	*0.497 (0.497-0.497)* ^b^	0.499 (0.499-0.499)	0.500 (0.500-0.500)
SVD^c^	0.498 (0.498-0.499)	0.499 (0.499-0.499)	0.499 (0.499-0.500)
SVD++	0.498 (0.498-0.499)	0.499 (0.499-0.499)	0.499 (0.499-0.499)

^a^kNN: k-nearest neighbor.

^b^Best result is italicized (per metric per trial).

^c^SVD: singular value decomposition.

#### Coverage

Tables 15 and 16 show the ISC of the kNN, SVD, and SVD++ algorithms when trained and tested on the hopefulness, similarity to the narrator, and similarity to the narrative ratings using NEON and NEON-O trial data, respectively.

For ISC, better means higher.

There was no better rating set, content-based filtering was the better approach for the NEON trial, there was no better approach for the NEON-O trial, and NEON-O was the better trial.

**Table 15:** Item space coverage (ISC; using Narrative Experiences Online [NEON] trial data).

ISC (NEON trial)	Hopefulness, median (IQR)	Similarity to the narrator, median (IQR)	Similarity to the narrative, median (IQR)
kNN^a^	*0.816 (0.816-0.816)* ^b^	0.811 (0.811-0.811)	0.800 (0.800-0.800)
SVD^c^	0.716 (0.711-0.721)	0.761 (0.759-0.764)	0.763 (0.763-0.764)
SVD++	0.709 (0.709-0.711)	0.757 (0.755-0.761)	0.771 (0.769-0.772)

^a^kNN: k-nearest neighbor.

^b^Best results is italicized (per metric per trial).

^c^SVD: singular value decomposition.

**Table 16:** Item space coverage (ISC; using Narrative Experiences Online for other [eg, nonpsychosis] mental health problems trial [NEON-O] trial data).

ISC (NEON-O trial)	Hopefulness, median (IQR)	Similarity to the narrator, median (IQR)	Similarity to the narrative, median (IQR)
kNN^a^	*0.891 (0.891-0.891)* ^b^	0.852 (0.852-0.852)	0.847 (0.847-0.847)
SVD^c^	0.848 (0.844-0.852)	0.840 (0.838-0.842)	0.847 (0.846-0.847)
SVD++	0.850 (0.849-0.851)	0.842 (0.841-0.843)	0.848 (0.844-0.852)

^a^kNN: k-nearest neighbor.

^b^Best results is italicized (per metric per trial).

^c^SVD: singular value decomposition.

#### Unfairness Across Participants

Tables 17 and 18 show the unfairness, based on gender, of the kNN, SVD, and SVD++ algorithms when trained and tested on the hopefulness, similarity to the narrator, and similarity to the narrative using NEON and NEON-O trial data, respectively.

For unfairness across participants based on gender, better means lower.

Hopefulness was the better rating set, collaborative filtering was the better approach, and there was no better trial.

Tables 19 and 20 show the unfairness, based on ethnicity, of the kNN, SVD, and SVD++ algorithms when trained and tested on the hopefulness, similarity to the narrator, and similarity to the narrative ratings using NEON and NEON-O trial data, respectively.

For unfairness across participants based on ethnicity, better means lower.

Hopefulness was the better rating set, collaborative filtering was the better approach, and there was no better trial.

**Table 17:** Unfairness across participants based on gender (using Narrative Experiences Online [NEON] trial data).

Unfairness (gender; NEON trial)	Hopefulness, median (IQR)	Similarity to the narrator, median (IQR)	Similarity to the narrative, median (IQR)
kNN^a^	0.429 (0.407-0.455)	0.715 (0.687-0.738)	0.728 (0.715-0.738)
SVD^b^	0.375 (0.347-0.422)	0.664 (0.637-0.695)	0.683 (0.669-0.688)
SVD++	*0.371 (0.342-0.412)* ^c^	0.673 (0.643-0.706)	0.695 (0.680-0.700)

^a^kNN: k-nearest neighbor.

^b^SVD: singular value decomposition.

^c^Best results are italicized (per metric per trial).

**Table 18:** Unfairness across participants based on gender (using Narrative Experiences Online for other [eg, nonpsychosis] mental health problems trial [NEON-O] trial data).

Unfairness (gender; NEON-O trial)	Hopefulness, median (IQR)	Similarity to the narrator, median (IQR)	Similarity to the narrative, median (IQR)
kNN^a^	0.387 (0.368-0.410)	0.727 (0.685-0.769)	0.765 (0.757-0.781)
SVD^b^	0.327 (0.312-0.344)	0.640 (0.634-0.652)	0.671 (0.659-0.696)
SVD++	*0.317 (0.303-0.338)* ^c^	0.647 (0.639-0.662)	0.685 (0.678-0.705)

^a^kNN: k-nearest neighbor.

^b^SVD: singular value decomposition.

^c^Best results are italicized (per metric per trial).

**Table 19:** Unfairness across participants based on ethnicity (using Narrative Experiences Online [NEON] trial data).

Unfairness (ethnicity; NEON trial)	Hopefulness, median (IQR)	Similarity to the narrator, median (IQR)	Similarity to the narrative, median (IQR)
kNN^a^	0.395 (0.370-0.417)	0.769 (0.757-0.783)	0.795 (0.776-0.808)
SVD^b^	0.345 (0.330-0.370)	0.732 (0.712-0.742)	0.727 (0.709-0.745)
SVD++	*0.338 (0.324-0.361)* ^c^	0.744 (0.722-0.755)	0.739 (0.716-0.763)

^a^kNN: k-nearest neighbor.

^b^SVD: singular value decomposition.

^c^Best results are italicized (per metric per trial).

**Table 20:** Unfairness across participants based on ethnicity (using Narrative Experiences Online for other [eg, nonpsychosis] mental health problems trial [NEON-O] trial data).

Unfairness (ethnicity; NEON-O trial)	Hopefulness, median (IQR)	Similarity to the narrator, median (IQR)	Similarity to the narrative, median (IQR)
kNN^a^	0.399 (0.378-0.421)	0.717 (0.687-0.754)	0.751 (0.724-0.787)
SVD^b^	*0.343 (0.328-0.350)* ^c^	0.652 (0.642-0.657)	0.667 (0.661-0.690)
SVD++	0.345 (0.331-0.353)	0.658 (0.649-0.664)	0.688 (0.680-0.708)

^a^kNN: k-nearest neighbor.

^b^SVD: singular value decomposition.

^c^Best results are italicized (per metric per trial).

#### MAE Over Time

Multimedia Appendix 6 shows how the median NMAE values changed over time (with an interval of 1 month) for the kNN, SVD, and SVD++ algorithms using “Hopefulness” ratings from NEON trial participants.

**Figure 1:**
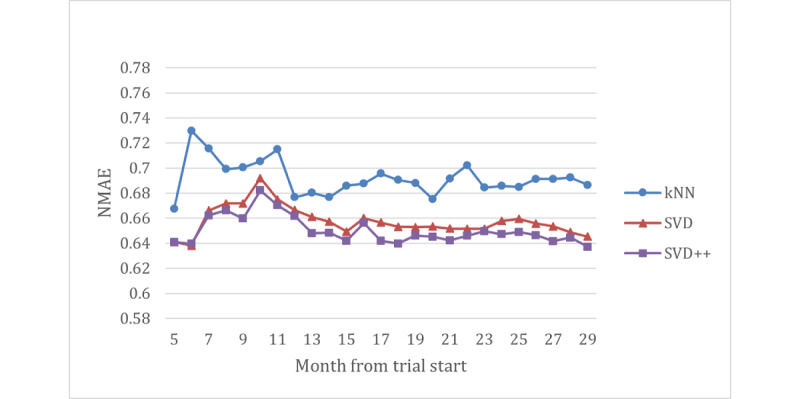
Comparison of the accuracy of the k-nearest neighbor (kNN), singular value decomposition (SVD), and SVD++ algorithms over time. NMAE: normalized mean absolute error.

Figure 1 shows that the 2 collaborative filtering algorithms were more accurate than the content-based filtering algorithm. As the number of ratings increases (and the IQR decreases), the NMAE stabilizes, which happens for all 3 algorithms at approximately 2000 ratings.

#### Other Results

Further analysis of the coverage metric showed that certain narratives were not routinely recommended by NarraGive, as described in Multimedia Appendix 7.

## Discussion

### Principal Findings

#### Overview

For the NEON trial, the content-based filtering algorithm performed better for coverage; the collaborative filtering algorithms performed better for accuracy, diversity, and unfairness across both gender and ethnicity; and neither algorithm performed better for precision. For the NEON-O trial, the content-based filtering algorithm did not perform better on any metric; the collaborative filtering algorithms performed better on accuracy and unfairness across both gender and ethnicity; and neither algorithm performed better for precision, diversity, or coverage. These findings provide preliminary evidence to inform future implementations.

Table 21 shows, for each metric, the filtering method that was better overall (per trial), the filtering method for the best result (per trial), and the trial in which all 9 values were better than those of the other trial. *N/A* indicates that neither trial nor filtering method was better overall. The table also indicates whether the metric was feature based or prediction based.

These results suggest that clinical population may be associated with recommender system performance. The content-based filtering algorithm had the best performance on feature-based metrics, suggesting that collaborative filtering methods may be associated with producing more accurate predictions whereas content-based filtering methods may be associated with recommending a wider range of items.

A low number of ratings for an item (or having only low ratings) can substantially influence how (or *if*) that item is recommended to other participants, as demonstrated by the 3 unrecommendable narratives in NarraGive.

**Table 21:** Summary of NarraGive evaluation metrics.

Metric	Prediction based or feature based	Better filtering method			Filtering method of best value			Better trial
		NEON^a^	NEON-O^b^	NEON		NEON-O		
NMAE^c^	Prediction	Collaborative	Collaborative	Collaborative		Collaborative	NEON-O	
Precision	Prediction	N/A^d^	N/A	Collaborative		Content	NEON	
ILD^e^	Feature	Collaborative	N/A	Collaborative		Content	NEON-O	
ISC^f^	Feature	Content	N/A	Content		Content	NEON-O	
Unfairness (gender)	Prediction	Collaborative	Collaborative	Collaborative		Collaborative	N/A	
Unfairness (ethnicity)	Prediction	Collaborative	Collaborative	Collaborative		Collaborative	N/A	

^a^NEON: Narrative Experiences Online.

^b^NEON-O: Narrative Experiences Online for other (eg, nonpsychosis) mental health problems trial.

^c^NMAE: normalized mean absolute error.

^d^N/A: not applicable.

^e^ILD: intralist diversity.

^f^ISC: item space coverage.

#### Unrecommendable Narratives

Each trial comprised between 1 and 3 ratings for each narrative. This preliminary evaluation only used data from NEON and NEON-O participants (to mirror the data used in the metrics). No rating given by a NEON or NEON-O participant was the highest, and only 1 rating had the optional questions answered (and none of these values were the highest value). The low number of ratings and the low scores given could contribute to the unrecommendableness of these narratives.

NarraGive, and recommender systems in general, often requires that there is a minimum amount of information about a participant or an item before being able to produce recommendations for or about them. In this case, due to SurPRISE’s implementation of recommender system algorithms, the kNN, SVD, and SVD++ algorithms require at least one rating for an item for it to be recommended and at least one rating by a participant for them to be recommended a narrative (to the extent that the recommender system never sees these unrated items and nonrating participants as they are filtered out before being passed to the recommender system).

This means that newly added narratives cannot be recommended immediately—they need at least one participant to access the narrative through another method (such as browsing to it) and then rate it. Consequently, there are other unrecommendable narratives (where unrecommendable in this case means that the recommender system does not have *access* to it in the first place rather than having access to it but not producing it as a recommendation for any participant). This is known as the *cold start problem*.

#### Interpretation of Metrics

In the context of NarraGive, diversity is not necessarily better or worse. An earlier substudy of the NEON study showed that there is unlikely to be a universally hopeful narrative [29], so a skew in recommended narratives is not necessarily a flaw.

Similarly, having greater coverage (ie, being able to recommend a greater proportion of available narratives) may not be useful if some narratives are only hope inspiring for a very small subset of participants.

The 2 unfairness metrics (unfairness across participants based on ethnicity and gender) capture 2 types of unfairness but not all. A previous NEON study [29] identified 7 harm minimization strategies for the NEON intervention, which provided the basis for the unfairness metric (ie, that being recommended narratives with a predicted rating that is higher than the resulting rating may be harmful). However, other types of unfairness, such as those based on disability, were not explored.

### Relationship to Prior Work

#### Recommender Systems for Nonnarrative Texts

Several book recommendation systems exist [59-61], but the focus has mostly been on novellike books rather than on health narratives or recovery narratives. In addition, many health recommender systems exist or have been proposed [62-72], but the focus has largely been on physical health and behavior changes rather than on providing desired content, such as enjoyable, useful, or hope-inspiring recovery narratives.

#### Dimensionality Reduction

The per-trial values for ILD were very similar. One explanation for this is a known phenomenon called the curse of dimensionality, where the increase in the number of dimensions (where the number of INCRESE characteristics represents the number of dimensions) causes a rapid increase in the “volume” that samples can occupy, which increases the data sparsity exponentially [73]. This increase in dimensionality produces effects such as the concentration of measure [74], where distance values converge and the difference between the furthest and the nearest point tends toward 0 [75], effectively making distance-based similarity comparisons meaningless. High-dimensionality problems can occur with as few as 10 dimensions [75], making the 77-item INCRESE measure susceptible to these issues. This may explain why the results for ILD are so similar—they are based on cosine distance. Other distance measures such as the Euclidean distance and correlation are also susceptible to this challenge [73].

One solution is to reduce the dimensionality of the characteristics before analysis through dimension reduction techniques [75] such as principal component analysis or matrix factorization, which retain as much of the original meaning of the data as possible while reducing the number of dimensions to a practical number.

### Strengths and Limitations

There are several strengths to this study. First, the NEON and NEON-O trials produced a unique data set of participant ratings, comprising ratings from both mental health service users and non–service users. This data set was suitable for analysis over time and for comparison of content-based and collaborative filtering algorithms.

Second, the numerous narrative request routes helped prevent exposure bias, and requiring a rating for each narrative helped prevent selection bias.

There are also some limitations to this study. This analysis did not consider individual participants’ rating patterns. Further analysis could add participant and item biases, which take into account items’ and participants’ average ratings to find the *deviation* from this average [76], or weight high-data participants (who have provided many ratings) as more informative than low-data participants.

The decision to include “no change” in hopefulness as an indicator of relevance was made to distinguish from actively hope-reducing narratives, but an alternative approach would be to only include those narratives rated as “a bit more hopeful” or “much more hopeful.”

The unfairness across participants metric (overestimation of unfairness) was based on the assumption that overestimated narratives are more likely to be harmful, but it is possible that a participant could rate a narrative highly and still find it harmful and, similarly, rate a narrative as lower than predicted but not find it harmful. The unfairness metrics also did not cover all aspects of unfairness.

Finally, this study is the first evaluation of a recommender system application to lived experience narratives. This is a complex area involving both technical challenges such as the choice of algorithm and ethical challenges such as managing narratives with respect and not just as another form of data. This complexity means that there are no existing standards against which NarraGive can be currently judged, and hence, the comprehensive evaluation presented in this study is primarily intended to be formative for the field rather than evaluative of NarraGive.

### Recommendations

There are 6 recommendations for researchers, intervention developers, recommender system developers, and health care professionals.

First, recommender systems with a focus on providing the greatest variety and widest range of content may benefit from using a content-based kNN algorithm, whereas recommender systems with a focus on predicting participants’ ratings most accurately may benefit more from the SVD or SVD++ algorithm. Recommender systems with a focus on both should implement a hybrid model with suitably weighted filtering algorithms.

Second, health care professionals should be aware of the unrecommendability of some items and not rely on recommendations to cover the entire search space.

Third, researchers and intervention developers should carefully decide which feature of the recommender system (ie, variety or accuracy) is most important and optimize the recommender system for a specific feature. Depending on what aspect of a recommender system is most important, different methods exist for optimizing for a specific metric, such as for diversity [43] and unfairness [46].

Fourth, platforms containing a recommender system should include other item access mechanisms (such as being able to browse through items) to prevent feedback loops where participants can only rate items that already have many high ratings while unrated items remain unrated and unrecommended [28]. This helps reduce the number of inaccessible narratives (because if the recommender system is the only access route, any unrecommendable narratives will be entirely inaccessible to participants), and developers could include a “random” access route (in addition to the recommender system access route) that is weighted toward these inaccessible narratives.

Fifth, recommender system developers should actively encourage the rating of new items, such as by suggesting them to participants or having a random button that is weighted toward new narratives. Alternatively, the narratives could be given an initial set of ratings before being published.

Sixth, initial studies should be conducted on proposed recommender systems to find the number of ratings required for the accuracy to stabilize; for NarraGive, this was approximately 2000 ratings. Because clinical population may be associated with recommender system performance, initial studies should also be used to inform clinical population selection.

### Implications for Future Work

Key future questions include whether a single or hybrid recommender system is optimal, a wider consideration of available algorithms and clarification of the rationale for selection, the rationale and timing of training and retraining the algorithm, and the identification of the most important metrics through which algorithmic performance should be evaluated.

For example, future studies investigating the use of recommender systems for recommending narratives could incorporate the similarity between participants and *narratives* by training filtering algorithms to recommend narratives with narrators that are either similar to or different from participants depending on (either implicit or explicit) participant preference.

### Conclusions

Clinical population may be associated with recommender system performance. The collaborative filtering algorithms were more accurate and less unfair than the content-based filtering algorithm. Recommender systems are susceptible to a wide range of biases, and it is important to mitigate these by providing enough data for the recommender system to start with (to prevent overfitting), ensuring that there are other ways of accessing items besides through the recommender system (to prevent a feedback loop between accessed items and recommended items), and encouraging participants to provide feedback on every item they interact with (to prevent participants from only providing feedback when they have strong opinions).

## Appendix

Multimedia Appendix 1 Definitions of the categories within the “Browse Stories” page of the Narrative Experiences Online intervention and screenshots of the web application.

Multimedia Appendix 2 Available options in the Personal Profile in the Narrative Experiences Online intervention.

Multimedia Appendix 3 Equations used for the normalized mean absolute error, precision, intralist diversity, item space coverage, and unfairness across participants.

Multimedia Appendix 4 Rationale for not using 5 of the evaluation categories.

Multimedia Appendix 5 Graphs showing the length of use of the Narrative Experiences Online (NEON) intervention comparing NEON and NEON for other (eg, nonpsychosis) mental health problems (NEON-O) trial participants.

Multimedia Appendix 6 Graphs showing the normalized mean absolute error over time for the k-nearest neighbor, singular value decomposition (SVD), and SVD++ algorithms.

Multimedia Appendix 7 Nonrecommendable narratives.

## Abbreviations

FEVR: framework for evaluating recommender systems
ILD: intralist diversity
INCRESE: Inventory of Characteristics of Recovery Stories
kNN: k-nearest neighbor
MAE: mean absolute error
NEON: Narrative Experiences Online
NEON-O: Narrative Experiences Online for other (eg, nonpsychosis) mental health problems
NMAE: normalized mean absolute error
RMSE: root-mean-square error
SurPRISE: Simple Python Recommendation System Engine
SVD: singular value decomposition

## Data Availability

Data will be available upon reasonable request, supervised by the study sponsor. Only anonymous and pseudonymous elements of the data sets used or analyzed during the study will be available. Research data will be available from the study sponsor until the end of the retention period. After the retention period, availability through the study sponsor or chief investigator may be at their discretion. Contact the study sponsor through Research@nottshc.nhs.uk citing Integrated Research Application System ID249015.
