# Supplementary material for: The Implementation of Recommender Systems for Mental Health Recovery Narratives: Evaluation of Use and Performance
Source: JMIR Ment Health. 2024 Mar 29;11:e45754. doi: 10.2196/45754 (PMC11015364; doi:10.2196/45754)
Supplement: Multimedia Appendix 1 [file mental_v11i1e45754_app1.pdf]

# Multimedia Appendix 1

*This is a Multimedia Appendix to a full manuscript published in the J Med Internet Res. For full copyright and citation information see http://dx.doi.org/10.2196/jmir.45754.*

| **Button** | **Definition** INCRESE item number : item value |
|---|---|
| Gender | |
| Female | 11:1 |
| Male | 11:2 |
| Other gender | 11:3 |
| Ethnicity | |
| Asian | 13:1 |
| African/Caribbean | 13:2 |
| Multiple ethnicities | 13:3 |
| White | 13:5 |
| Other ethnicity | 13:4 |
| Sexuality | |
| Bisexual | 16:1 |
| Gay woman | 16:4 |
| Gay man | 16:2 |
| Heterosexual | 16:3 |
| Other sexuality | 16:5 |
| Recovery status | |
| Rejects recovery | 34:5 |
| Surviving | 34:3 |
| Making progress | 34:2 |
| Living well | 34:1 |
| Recovered | 34:4 |
| Diagnosis | |
| Uses non-diagnostic framework | 30:1 |
| Psychosis | 27:1 |
| Mood related | 24:1 |
| Developmental related | 22:1 |
| Eating related | 23:1 |
| OCD | 26:1 |
| Stress or PTSD | 28:1 |
| Substance problems | 29:1 |
| Personality related | 25:1 |
| Contains | |
| Text | 5:1 |
| Audio | 6:1 |
| Moving images | 7:1 |
| Static images | 8:1 |
| Tone | |
| Upbeat | 33:1 |
| Downbeat | 33:2 |
| Critical | 33:3 |
| Neutral | 33:4 |
| Recovery is | |
| Despite services | 32:2 |
| Outside services | 32:3 |
| Within services | 32:1 |
| Genre | |
| Endurance | 31:2 |
| Endeavour | 31:3 |
| Escape | 31:1 |
| Enlightenment | 31:4 |
| A positive story | 33.1 AND (31.1 OR 31.4) |
| A shorter story | (9>0 AND <300) OR (10>0 AND <5) |

*Table S1. Definitions of the categories on the "Browse Stories" page, defined using narratives' INCRESE characteristics.*

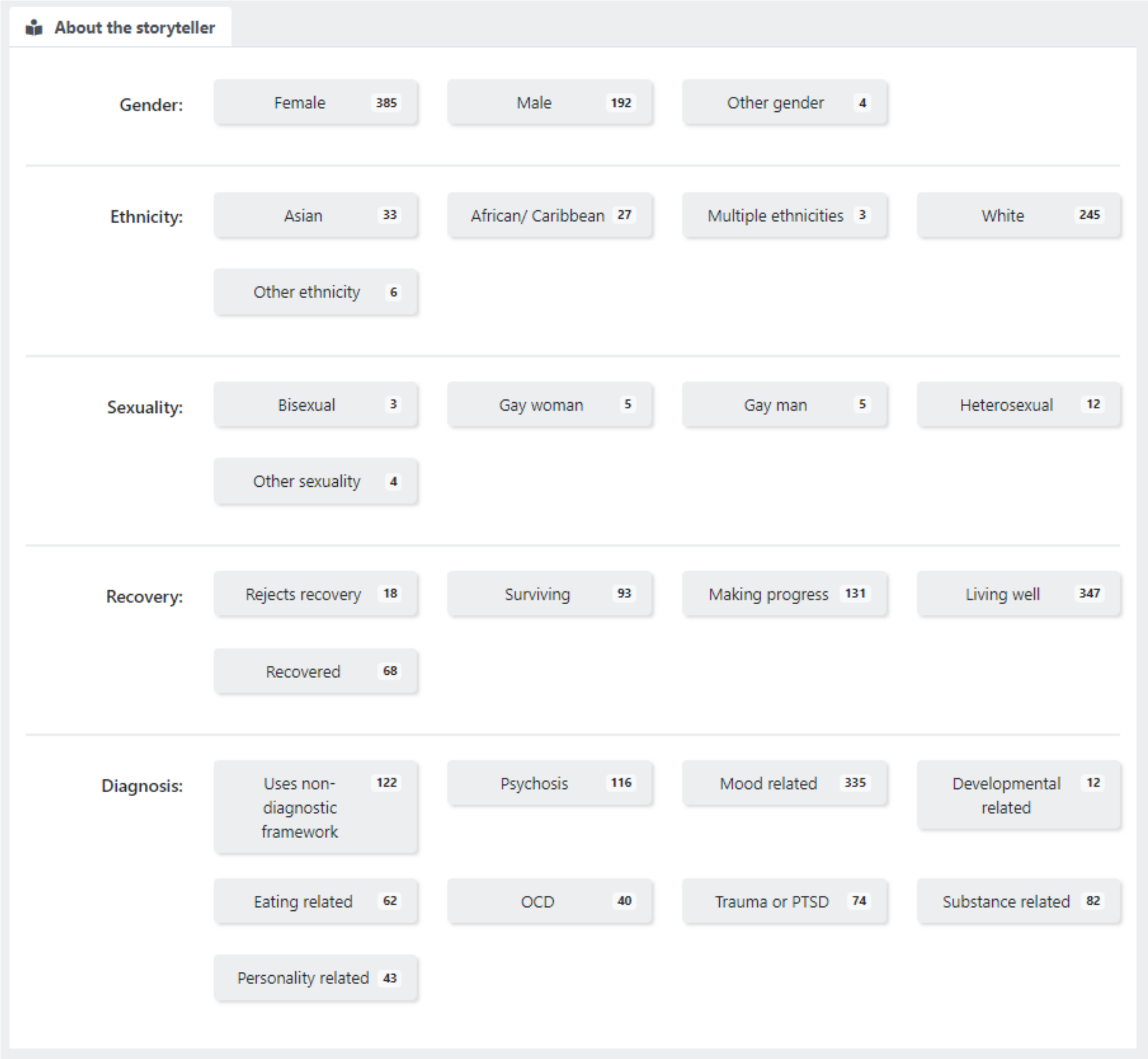


*Figure S1. Categories relating to the storyteller, displayed on the "Browse Stories" page.*

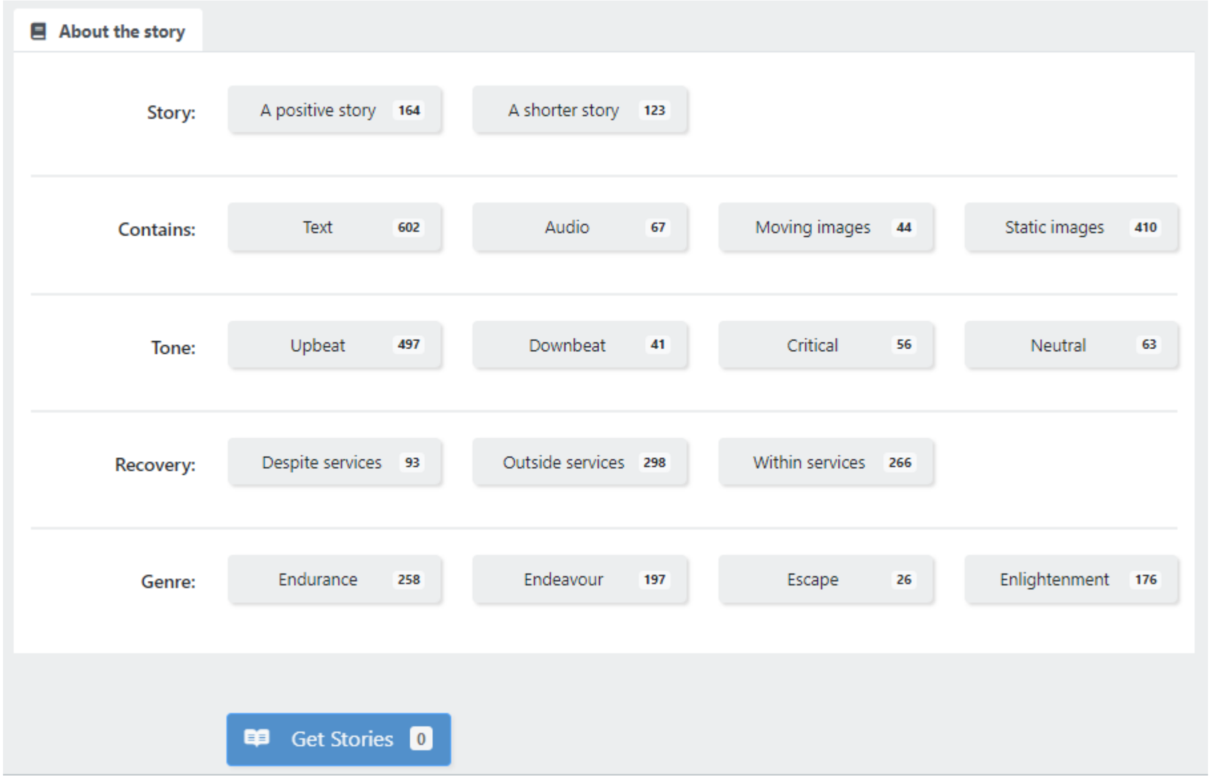


*Figure S2. Categories relating to the story, displayed on the "Browse Stories" page.*
