# Supplementary material for: The Implementation of Recommender Systems for Mental Health Recovery Narratives: Evaluation of Use and Performance
Source: JMIR Ment Health. 2024 Mar 29;11:e45754. doi: 10.2196/45754 (PMC11015364; doi:10.2196/45754)
Supplement: Multimedia Appendix 3 [file mental_v11i1e45754_app3.pdf]

Multimedia Appendix 3

This is a Multimedia Appendix to a full manuscript published in the J Med Internet Res. For full copyright and citation information see http://dx.doi.org/10.2196/jmir.45754.

Prediction accuracy

Given a test set T of participant-narrative pairs, let r_{pn} denote the normalized true rating of participant p for narrative n, and $\hat{r}_{pn}$ denote NarraGive's predicted rating for p and n.

$$\mathrm{NMAE} = \frac{1}{|\mathrm{T}|} \sum_{(\mathrm{p,n}) \in \mathrm{T}} \left| \hat{\mathrm{r}}_{\mathrm{pn}} - \mathrm{r}_{\mathrm{pn}} \right|$$

Equation S1 - Prediction accuracy equation: NMAE.

Usage prediction

For a participant p and narrative n, let r_{pn} be p's true rating for n. Define relevance for r, $relevant(r)$, as:

$$\mathrm{relevant(r)} = \begin{cases} True & if\ r \geq 1 \\ False & otherwise \end{cases}$$

(This uses the normalized rating sets; in the un-normalized rating sets, a hopefulness rating is relevant when it is greater than or equal to *0*)

Define p's set of relevant narratives, N_p, as:

$$N_p = \{n: relevant(r_{pn})\}$$

Let R_p be p's recommendations from NarraGive. Define precision as:

$$\mathrm{precision}(p) = \frac{|R_p \cap N_p|}{|R_p|}$$

Thus given the set of participants P:

$$\mathrm{MAPP} = \frac{1}{|P|} \sum_{p \in P} \mathrm{precision}(p)$$

Equation S2 - Usage prediction equation: precision.

Diversity

Define cosine similarity for two narratives, i and j, as the cosine of the angle between a vector of i's INCRESE characteristics, $\vec{\imath}$, and a vector of j's INCRESE characteristics, $\vec{\jmath}$:

$$\mathrm{cossim}(i,j) = \frac{\vec{\imath} \cdot \vec{\jmath}}{\|\vec{\imath}\| \|\vec{\jmath}\|}$$

The ILD of participant p's recommendation list R_p is defined as:

$$\mathrm{ILD}(p) = \sum_{i \in R_p} \sum_{j \in R_p, i \neq j} \mathrm{cossim}(i,j)$$

Thus given the set of participants P, the overall ILD is calculated as:

$$\mathrm{ILD} = \frac{1}{|P|} \sum_{p \in P} \mathrm{ILD}(p)$$

Equation S3 - Diversity equation: ILD.

Coverage

Let N denote the set of all narratives, P the set of all participants, and N_p the set of 10 internal recommendations from NarraGive for participant p.

$$\mathrm{ISC} = \frac{\left|\bigcup_{p \in P} N_p\right|}{|N|}$$

Equation S4 - Coverage equation: ISC.

Unfairness across participants

Overestimation of unfairness is defined as:

Let the set of participants P be indexed from 1 to m, and the set of items from 1 to n. Let y_{ij} be the predicted rating for the ith participant and jth item, r_{ij} the true rating of i for j, X the set of true participant-item rating pairs, and $g \subseteq P$ the set of disadvantaged participants. Let d denote the set of disadvantaged participants who have made at least one rating, computed as:

$$d = \{i : ((i, j) \in X) \land i \in g\}$$

Let $E_g[y]_j$ denote the average predicted rating from disadvantaged participants for the jth item, computed as:

$$E_g[y]_j = \frac{1}{|d|} \sum_{i \in d} y_{ij}$$

The average predicted rating from non-disadvantaged participants for j, $E_{\neg g}[y]_j$, and the average true rating from disadvantaged and non-disadvantaged participants for j, $E_g[r]_j$ and $E_{\neg g}[r]_j$ respectively, are computed analogously.

Define overestimation of unfairness as:

$$\mathrm{OU} = \frac{1}{n} \sum_{j=1}^{n} \left| \max\{0, E_g[y]_j - E_g[r]_j\} - \max\{0, E_{\neg g}[y]_j - E_{\neg g}[r]_j\} \right|$$

Equation S5 - Unfairness across participants equation: OU.
