# Supplementary material for: The Implementation of Recommender Systems for Mental Health Recovery Narratives: Evaluation of Use and Performance
Source: JMIR Ment Health. 2024 Mar 29;11:e45754. doi: 10.2196/45754 (PMC11015364; doi:10.2196/45754)
Supplement: Multimedia Appendix 4 [file mental_v11i1e45754_app4.pdf]

Multimedia Appendix 4

This is a Multimedia Appendix to a full manuscript published in the J Med Internet Res. For full copyright and citation information see http://dx.doi.org/10.2196/jmir.45754.

Five categories of the Zangerie and Bauer [41] framework were not used.

Ranking

Within the NEON Intervention web application, there are four ways of accessing narratives: a single recommendation provided by NarraGive, a single narrative selected at random, a list of narratives filtered by tags, and a list of previously bookmarked or hopeful narratives.

Ranking metrics measure the quality of a ranked list of recommendations [41], but since NarraGive presents a single recommendation rather than a list, this category of metrics was not used.

Novelty

Novelty metrics measure the extent to which recommended items are new to the participant. However, since NarraGive was designed to only recommend un-accessed narratives, this category of metrics was not used.

Serendipity

Serendipity, similar to novelty, measures how new and *unexpected* recommendations are [33]. Since this element of surprise is provided through the random-access method instead, this category of metrics was not used.

Fairness

Fairness across items, analogous to unfairness across participants, measures the fairness of exposure across item groups [41]. This category of metrics is based on the assumption that equally-relevant items should have equal exposure [57]. However, given the highly personal nature of health experiences, it is less important whether there is variation in exposure of similarly-relevant items, and more important that the items that *are* presented are relevant (measured by the precision metric), that there is fairness across *participants* (measured by the OU metric), and that there is *overall* exposure of items (measured by the ISC metric). Thus, this category of metrics was not used.

Business-oriented

Business-oriented metrics measure the business value of recommendations [41]. Since NarraGive is not used in a business context (ie, the authors of the narratives neither pay to have their narratives recommended, nor receive payment for usage of their narratives; the owners of the web application do not receive payment when participants use the site or rate narratives highly; and there are no items available for sale), this category of metrics was not used.
