# Supplementary material for: The Implementation of Recommender Systems for Mental Health Recovery Narratives: Evaluation of Use and Performance
Source: JMIR Ment Health. 2024 Mar 29;11:e45754. doi: 10.2196/45754 (PMC11015364; doi:10.2196/45754)
Supplement: Multimedia Appendix 5 [file mental_v11i1e45754_app5.pdf]

Multimedia Appendix 5

This is a Multimedia Appendix to a full manuscript published in the J Med Internet Res. For full copyright and citation information see http://dx.doi.org/10.2196/jmir.45754.

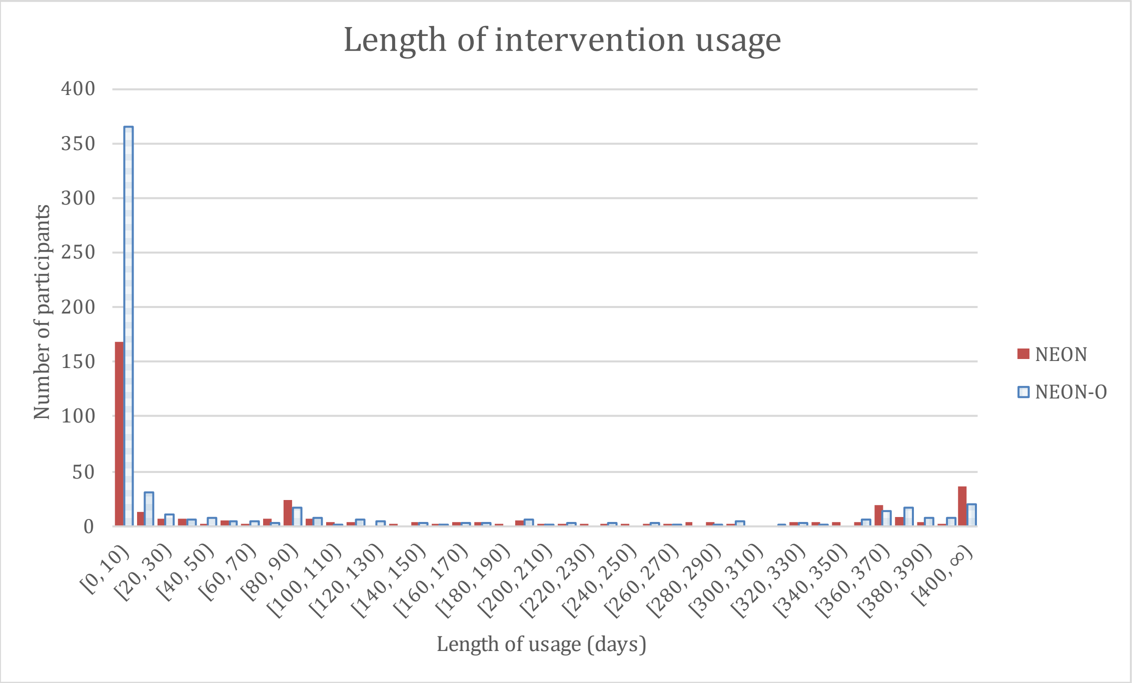

Figure S1 - Length of time between first and last narrative request, for both NEON and NEON-O participants.

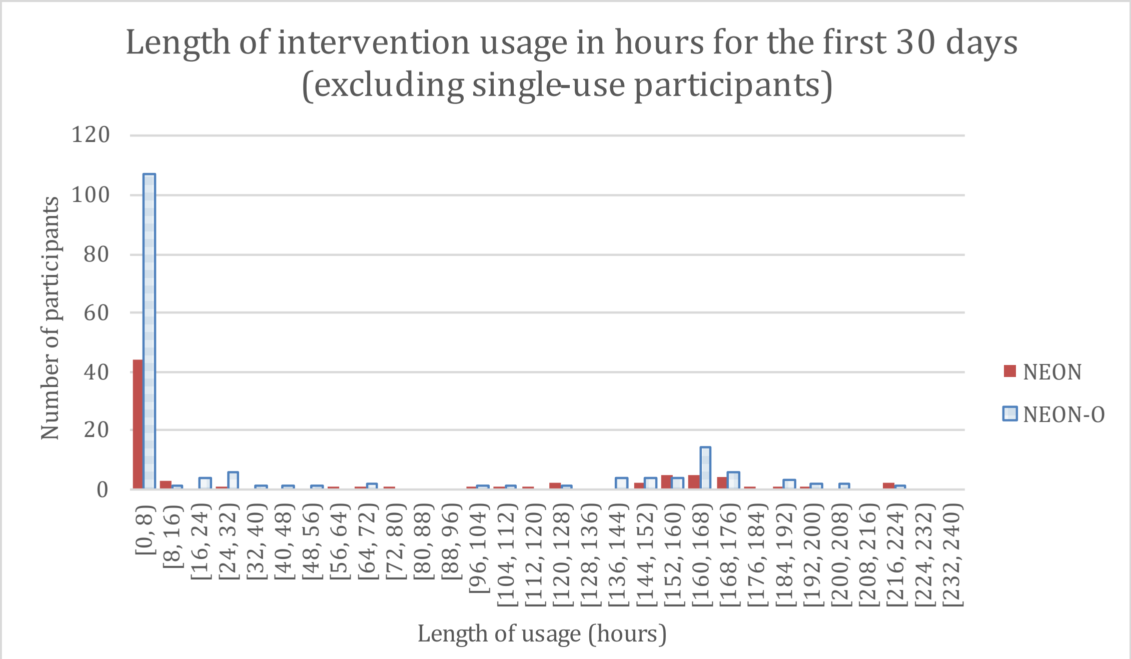

Figure S2 - Length of time between first and last narrative request showing only lengths up to thirty days, excluding participants who only used the intervention once, for both NEON and NEON-O participants.
