# Supplementary material for: The Implementation of Recommender Systems for Mental Health Recovery Narratives: Evaluation of Use and Performance
Source: JMIR Ment Health. 2024 Mar 29;11:e45754. doi: 10.2196/45754 (PMC11015364; doi:10.2196/45754)
Supplement: Multimedia Appendix 6 [file mental_v11i1e45754_app6.pdf]

Multimedia Appendix 6

This is a Multimedia Appendix to a full manuscript published in the J Med Internet Res. For full copyright and citation information see http://dx.doi.org/10.2196/jmir.45754.

This appendix contains graphs showing the Normalised Mean Absolute Error (NMAE) over time for kNN, SVD and SVD++ algorithms.

kNN

Figure S1 shows how the median NMAE value changes over time (with an interval of one month) for the kNN algorithm, using 'Hopefulness' ratings for NEON Trial participants.

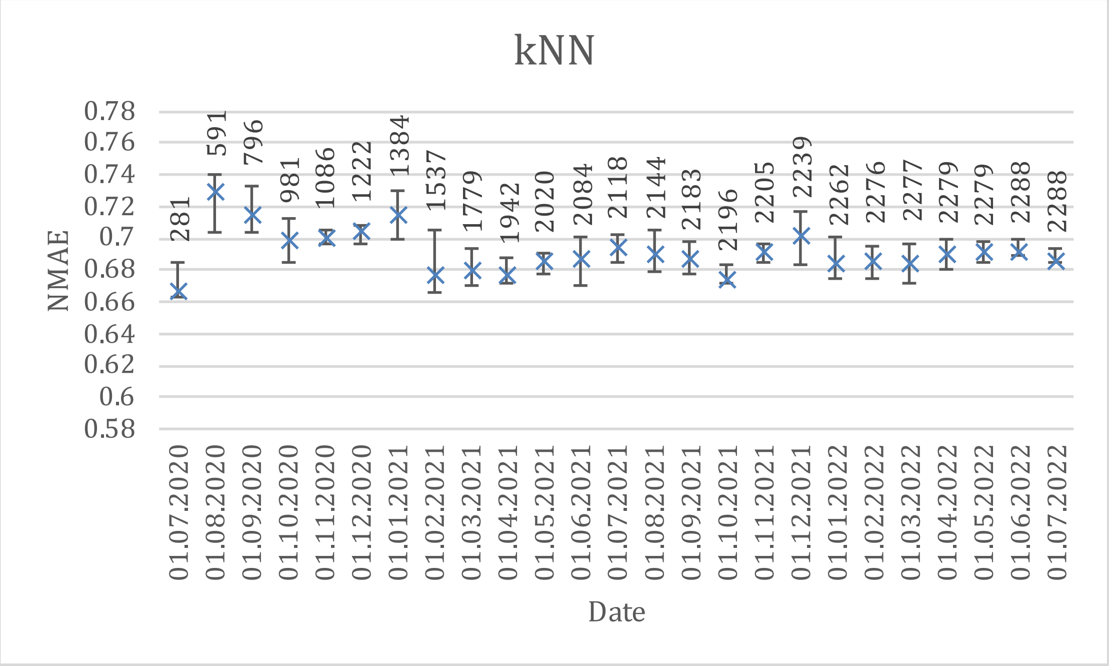

Figure S1 - The accuracy of the kNN algorithm, measured using NMAE, over time when trained on data up to and including the specified month. Error bars show the IQR, and data labels show the number of ratings available for training and testing.

SVD

Figure S2 shows how the median NMAE value changes over time (with an interval of one month) for the SVD algorithm, using 'Hopefulness' ratings for NEON Trial participants.

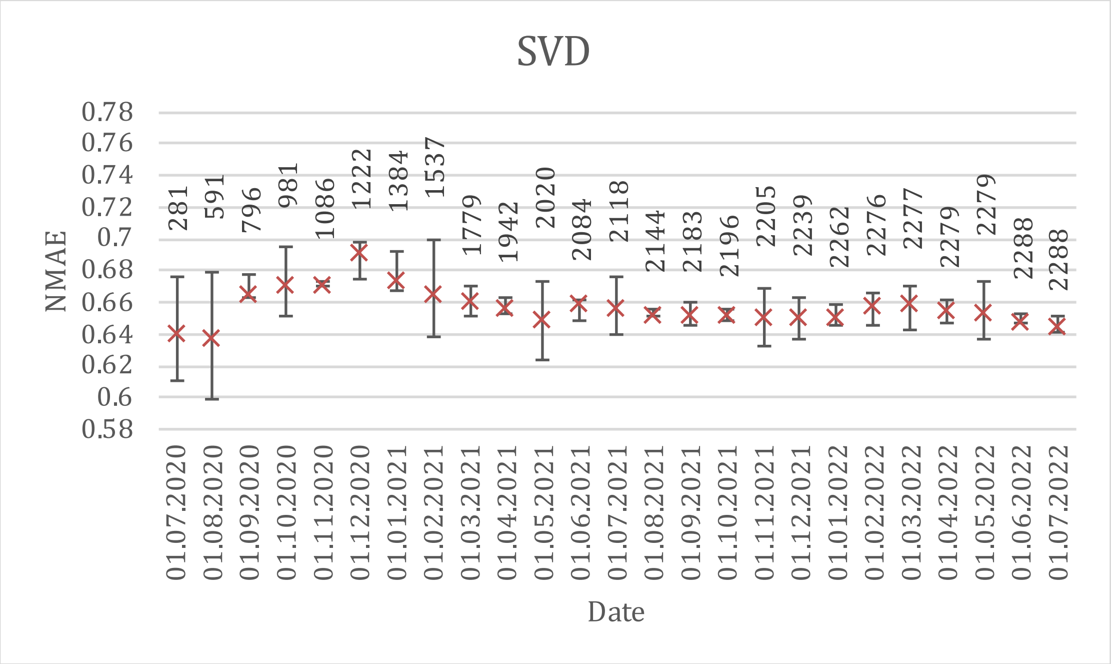

Figure S2 - The accuracy of the SVD algorithm, measured using NMAE, over time when trained on data up to and including the specified month. Error bars show the IQR, and data labels show the number of ratings available for training and testing.

SVD++

Figure S3 shows how the median NMAE value changes over time (with an interval of one month) for the SVD++ algorithm, using 'Hopefulness' ratings for NEON Trial participants.

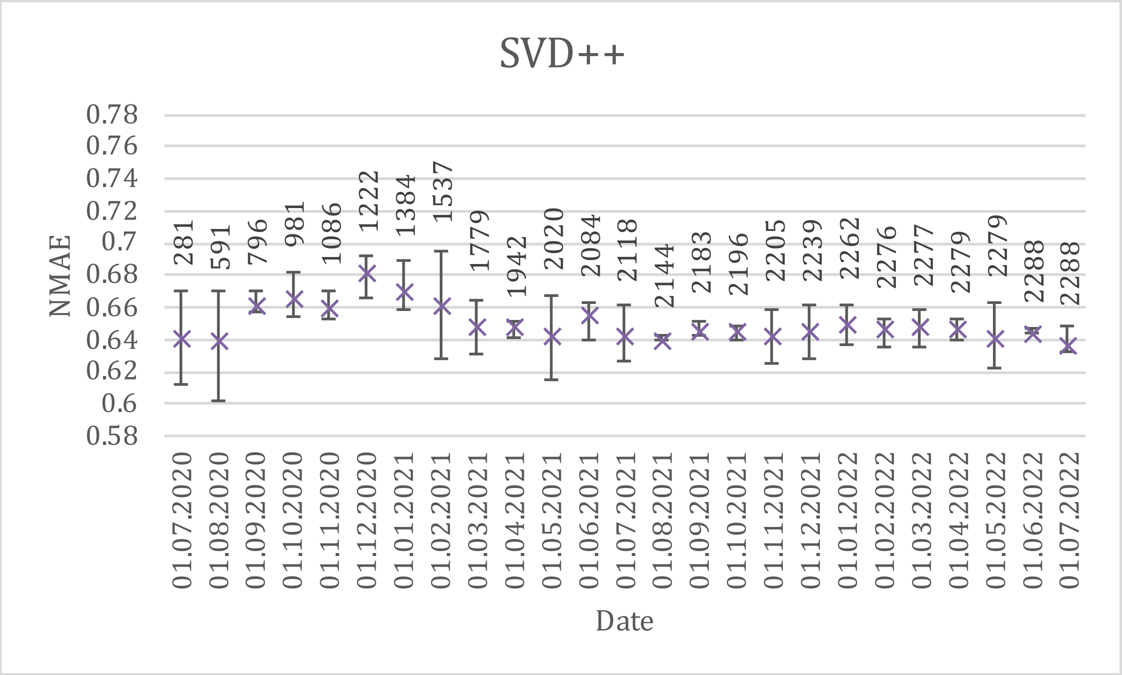

Figure S3 - The accuracy of the SVD++ algorithm, measured using NMAE, over time when trained on data up to and including the specified month. Error bars show the IQR, and data labels show the number of ratings available for training and testing.
