# Supplementary material for: The Implementation of Recommender Systems for Mental Health Recovery Narratives: Evaluation of Use and Performance
Source: JMIR Ment Health. 2024 Mar 29;11:e45754. doi: 10.2196/45754 (PMC11015364; doi:10.2196/45754)
Supplement: Multimedia Appendix 7 [file mental_v11i1e45754_app7.pdf]

Multimedia Appendix 7

This is a Multimedia Appendix to a full manuscript published in the J Med Internet Res. For full copyright and citation information see http://dx.doi.org/10.2196/jmir.45754.

It was found that three narratives could not be recommended (i.e., did not appear in NarraGive's top-10 list for any participant) when NarraGive was trained on any combination of NEON Trial data, NEON-O Trial data, Hopefulness rating set, either of the two Similarity rating sets, and any of the three filtering algorithms.

Accesses to narratives 1, 2 and 3 are summarized in Table S1.

**Narrative**	**Hope**	**Similarity to Narrator**	**Similarity to Narrative**	**Learning**	**Empathy**	**Trial**
1	0	Not given	Not given	Not given	Not given	NEON
1	-1	Not given	Not given	Not given	Not given	NEON-O
1	-1	Not given	Not given	Not given	Not given	NEON-O
1	1	Not given	Not given	Not given	Not given	NEON
2	1	Not given	Not given	Not given	Not given	NEON
2	0	Not given	Not given	Not given	Not given	NEON-O
3	1	1	1	1	1	NEON-O
3	0	Not given	Not given	Not given	Not given	NEON-O
3	1	Not given	Not given	Not given	Not given	NEON-O
3	0	Not given	Not given	Not given	Not given	NEON

Table S1 - Narrative ratings for the three unrecommendable narratives using synthetic narrative IDs to avoid identification.

Each row represents a rating given by a participant. The table shows all ratings for the three narratives.
